# Aberrant Apoptotic Response of Colorectal Cancer Cells to Novel Nucleoside Analogues

**DOI:** 10.1371/journal.pone.0138607

**Published:** 2015-09-21

**Authors:** Leonie Harmse, Nurit Dahan-Farkas, Jenny-Lee Panayides, Willem van Otterlo, Clement Penny

**Affiliations:** 1 Division of Pharmacology, Department of Pharmacy and Pharmacology, Faculty of Health Sciences, University of the Witwatersrand, 7 York Road, Parktown, 2193, South Africa; 2 Molecular Sciences Institute, School of Chemistry, Faculty of Science, University of the Witwatersrand, Private Bag 3, Johannesburg 2050, South Africa; 3 Department of Chemistry and Polymer Sciences, Stellenbosch University, Private Bag XI, Matieland 7602, South Africa; 4 Department of Internal Medicine, Faculty of Health Sciences, University of the Witwatersrand, 7 York Road, Parktown 2193, South Africa; University of Pecs Medical School, HUNGARY

## Abstract

Despite the increased understanding of colorectal cancer and the introduction of targeted drug therapy, the metastatic phase of the disease remains refractory to treatment. Since the deregulation of normal apoptosis contributes to the pathogenesis of colorectal cancer, novel nucleoside analogues were synthesized here and evaluated for their ability to induce apoptosis and cause cell death in two colorectal adeno-carcinoma cell lines, Caco-2 and HT-29. Three novel nucleoside analogues assessed here showed cytotoxic activity, as measured by the MTT assay against both cell lines: the IC_50_ values ranged between 3 and 37 μM, with Caco-2 cells being more sensitive than HT-29 cells. Compared to camptothecin, the positive control, the nucleoside analogues were significantly less toxic to normal unstimulated leukocytes (*p*>0.05). Moreover, the nucleosides were able to induce apoptosis as measured by an increase in caspase 8 and caspase 3 activity above that of the control. This was additionally supported by data derived from Annexin V-FITC assays. Despite marginal changes to the mitochondrial membrane potential, all three nucleosides caused a significant increase in cytosolic cytochrome c (*p*>0.05), with a corresponding decrease in mitochondrial cytochrome c. Morphological analysis of both cell lines showed the rapid appearance of vacuoles following exposure to two of the nucleosides, while a third caused cellular detachment, delayed cytoplasmic vacuolisation and nuclear abnormalities. Preliminary investigations, using the autophagic indicator monodansylcadaverine and chloroquine as positive control, showed that two of the nucleosides induced the formation of autophagic vacuoles. In summary, the novel nucleoside analogues showed selective cytotoxicity towards both cancer cell lines and are effective initiators of an unusual apoptotic response, demonstrating their potential to serve as structural scaffolds for more potent analogues.

## Introduction

There has been substantial progress in the understanding of the molecular mechanisms underlying colorectal cancer. However, despite the use of targeted drug therapy, colorectal cancer remains associated with a poor prognosis. While drugs like bevacizumab have improved the survival period for metastatic disease beyond 20 months, they have not been able to affect a cure. Recently, clinical trials have demonstrated the failure of tyrosine kinase inhibitors such as sunitinib, which caused an increase in severity and incidence of serious adverse drug reactions with minimal therapeutic benefit [1]. Therefore, the search for effective agents to treat advanced colorectal cancer remains a priority.

Numerous studies have shown that the normal apoptotic process is dysfunctional in colorectal cancer [2–5]. Dysregulation of apoptosis contributes to the development of resistance to chemotherapy and a poor prognosis [6]. Apoptosis, as first described by Kerr in 1972 [7], is characterised by a number of morphological changes, including plasma membrane blebbing, chromatin condensation, nuclear fragmentation and formation of apoptotic bodies. Hallmarks of apoptosis include the exposure of phosphatidylserine on the outer leaflet of the plasma membrane [8, 9], changes to mitochondrial membrane permeability [10] and the release of cytochrome c from the mitochondrial inter-membrane space, with the subsequent activation of effector caspases [11].

Apoptosis is essential for the regulation of normal cell turnover of the intestinal epithelium. However, in colorectal cancer, both the extrinsic and the intrinsic apoptotic pathways are compromised favouring the proliferation of neoplastic cells. In colorectal cancer, despite FAS receptor expression, cells are resistant to FAS ligand mediated apoptosis indicating a defect in FAS mediated signalling [12]. TNF related apoptosis inducing ligand (TRAIL) receptor mediated apoptosis is normally upregulated in cancer cells; however, in colorectal cancer, resistance to TRAIL receptor mediated apoptosis has been observed [13]. The p53 tumour suppressor gene product is mutated or absent in 85% of colorectal cancers. This transcription factor is essential for the activation of the intrinsic apoptotic pathway as well as the expression of the cyclin dependent kinase inhibitor p21^waf1/Cip1^. Cells that are defective in p53 have decreased levels of apoptotic proteins, thereby favouring the proliferation of neoplastic cells [14].

Chemotherapeutic agents that are able to induce apoptosis are beneficial, since they bring about death of cancer cells without triggering the inflammatory response and the tumour lysis syndrome associated with necrosis. Nucleoside analogues are used extensively to treat cancer and manage viral infections like Herpes and HIV. Drugs belonging to this class are known to induce apoptosis in the cancers where they are effective [15].

In the present study, novel nucleoside analogues were screened against two colon cancer cell lines, to investigate both their cytotoxic effects and their potential to induce apoptosis. We showed that despite showing relatively high IC_50_ values as determined by the MTT cytotoxicity assay, some nucleoside analogues were comparable to camptothecin with regards to their ability to induce apoptosis. Furthermore the compounds induced an unusual mitochondrial response which may provide further clues to the underlying apoptotic abnormalities in colorectal cancer.

## Materials and Methods

### Cell culture

HT-29 (HTB-38™) and Caco-2 (HTB-37™) human colorectal cancer cell lines obtained from ATCC, were cultured in low glucose Dulbecco’s Modified Eagle’s Medium (DMEM) which contained 5% heat-inactivated foetal calf serum. Culture medium and foetal calf serum were obtained from Highveld Biologicals, South Africa. Cells were grown in 75 cm^2^ culture flasks and incubated at 37°C in a humidified atmosphere with 5% CO_2_. Both cell lines were subcultured when they reached confluence. Briefly, cells were washed twice with 10 ml phosphate buffered saline followed by the addition of 4 ml trypsin and incubation at 37°C for 10 minutes. Detached cells were reseeded to the same flask. Camptothecin (Sigma), a known inducer of apoptosis, and an inhibitor of topoisomerase-1, was used as a positive control in all experimental procedures.

### Assessment of cell viability

Assessment of test nucleoside cytotoxicity was carried out by the 3-(4,5-dimethylthiazol-2-yl)-2,5-diphenyltetrazolium bromide, (MTT), assay (Sigma). The Trypan Blue (Sigma) exclusion assay was used to assess the viability of the cancer cells prior to plating cells for nucleoside cell viability assays. Cells were seeded in 96 well plates at a cell density of 9 000 cells/well, allowed to adhere to the growth surface for 4 hours and then exposed to the test nucleosides for 48 hours at concentrations ranging between 1 and 120 μM. The MTT assay was essentially performed as described by Mossman [16] with the exception of dissolving the formazan crystals in DMSO. Briefly, following incubation with the MTT, 100 μl of media was replaced with DMSO to facilitate the solubilisation of the formazan crystals. The absorbance of the solubilised formazan crystals was measured at 540 and 690 nm with a Labsystems iEMS plate reader. Data was obtained from experiments that were repeated at least three times, with each data point being determined in quadruplicate. Sigmoidal dose response curves were constructed with GraphPad Prism, version 5.

### Isolation of human peripheral leukocytes

Permission to isolate human leukocytes from healthy volunteers was obtained from the Human Ethics Committee of the Faculty of Health Sciences, University of the Witwatersrand (clearance number, M070519). Written, informed consent from each donor was obtained for the experimental use of her/his leukocytes in research. Peripheral leukocytes from a single donor were isolated from blood collected in a heparinised tube. Red blood cells were selectively lysed by using the Versagene Blood DNA Kit (Gentra Systems). Freshly prepared leukocytes were used to determine nucleoside toxicity to normal cells. The leukocytes were transferred to RPMI (Highveld Biologicals) culture medium supplemented with 2 mM glutamine (Sigma) and 10% foetal calf serum (Separations). Following the harvesting of the leukocytes, their viability was determined by the Trypan Blue exclusion assay before they were exposed to the test nucleosides for 24 hours.

### Assessment of apoptosis

#### Induction of caspase 3 and caspase 8 activity

Caspase 3 and caspase 8 specific colorimetric assays, namely CPP32 and FLICE, were used to measure caspase activity as per the manufacturer’s protocol (Biovision Research Products). The level of caspase activity in the cell lysates was directly proportional to the absorbance of *p*-nitroaniline (pNA) measured at 405 nm in a Labsystems iEMS Multiscan plate reader. Cells were seeded in 6 well plates at a density of 50 000 cells per well and allowed grow for 18 hours before treatment with nucleosides. The protein content was standardised in all test samples. Caspase 3 and caspase 8 activity was measured following different exposure periods of the cells to the test nucleosides, typically at 2, 4, 8, 12, and 24 hours after the addition of the nucleosides to the cultured cells.

#### Measurement of the mitochondrial membrane potential

The cationic dye 5,5’,6,6’-tetrachloro-1,1’,3,3’-tetraethylbenzimidazolcarbocyanine iodide (JC-1) is selective for mitochondria and its inherent fluorescence makes it useful to measure the electrochemical gradient that exists across the mitochondrial membrane. The protocol was followed as per the manufacturer’s specifications (BD Pharmingen). The JC-1 dye accumulates in the mitochondria of healthy cells as fluorescent red aggregates. Cells were seeded in 6 well plates at a density of 50 000 cells per well and allowed grow to near confluency before treatment with nucleosides. Flow cytometry, with excitation/emission filters of 485/540 nm (green fluorescence) and 540/590 nm (red fluorescence), was used to measure the effect of the test nucleosides on mitochondrial membrane potential. The mitochondria containing red JC-1 aggregates in healthy cells were detected in the FL-2 channel (upper gate in the scattergraphs), whereas green JC-1 monomers in apoptotic cells were detected in the FL-1 channel (lower gate in the scattergraphs).

#### Measurement of mitochondrial and cytoplasmic cytochrome c

The human cytochrome c Titerzyme Enzyme Immunometric Assay (Assay Designs Inc.) was used to measure the concentration of cytochrome c in the mitochondrial and the cytoplasmic fractions of cells exposed to the test nucleosides. Cells were seeded in 6 well plates at a density of 50 000 cells per well and allowed grow for 18 hours before treatment with nucleosides. The treated cells were harvested and rinsed with ice-cold phosphate buffered saline. The cytosolic fraction was obtained by resuspending the cell pellet with a cell permeabilization buffer, (250 mM sucrose, 137 mM NaCl, 70 mM KCl, 4.3 mM Na_2_HPO_4_, 1.4 mM K_2_HPO_4_, 0.2 mg/ml Digitonin and 0.1% Hydorol M) supplied in the Mitochondrial isolation kit, (Assay Designs Inc.) which left the mitochondria intact. This was followed by centrifugation to separate the cytosolic fraction and to pellet the mitochondria. The mitochondria were then lysed with RIPA buffer, consisting of 50 mM Tris-HCl, pH 7.4, 150 mM NaCl, 1mM EDTA, 1mM EGTA, 1% Triton X-100, 1% sodium deoxycholate and 0.1% SDS.

#### Determination of apoptosis as measured by the annexin V assay

Phosphatidylserine (PS) translocation to the outer cell membrane was determined by fluorescein isothiocyanate (FITC)-labelled Annexin V. Flow cytometric analyses with FITC-labelled annexin V and propidium iodide were performed according to the manufacturer’s protocol (BD Pharmingen), on cells exposed to 100 μM of each of the test nucleosides or camptothecin for 24 hours. Cells were seeded in 6 well plates at a density of 50 000 cells per well and allowed to grow to near confluency before treatment with nucleosides. Cell staining was assessed using Annexin V-FITC (green fluorescence) as well as a dye exclusion of propidium iodide (PI) (negative for red fluorescence), recording at least 10^4^ events. Following exposure to the test nucleosides, the cells were harvested and analysed by flow cytometry on a BD LSR II flow cytometer. The data was plotted as a scattergraph with FITC-Annexin V (green fluorescence) represented on the X-axis *versus* PI (red fluorescence) on the Y-axis.

#### Caspase 9 activity

Caspase 9 activity was determined with the Abcam® Caspase 9 active FITC staining kit. The caspase 9 selective inhibitor LEHD-FMK conjugated to FITC penetrates live cells to bind to active caspase 9 in an irreversible manner. Cells were seeded on sterile coverslips (50 000 per coverslip) allowed to adhere for four hours and exposed to the test nucleosides and camptothecin, respectively, for 24 hrs. Thereafter cells were washed with PBS and then incubated with the substrate at 37°C for one hour. Slides were washed in PBS and viewed on an Olympus BX41 epifluorescence microscope. Images were captured with an Olympus DP72 camera and analysed with the Olympus CellSens Software package. Cells with activated Caspase 9 display a bright green fluorescence.

### Assessment of HT-29 and Caco-2 cell morphology

The effect of the nucleosides on cell morphology was assessed by phase contrast and fluorescence microscopy. For phase contrast microscopy, cells were grown in 6 well culture plates (50 000 cells per well), allowed to adhere overnight and then exposed to nucleosides for various time periods. All experiments were repeated at least three times. Cells were observed with an Olympus CKX41 inverted microscope and images were captured with an Olympus DP21 camera. Cell morphology was further evaluated with the Hoechst 33342 (Life Technologies) and acridine orange (Life Technologies) fluorescent dyes. Cells were cultured on heat sterilised glass coverslips and exposed to the test nucleosides at varying concentrations. Using the appropriate filters, cells were observed with an Olympus BX41 epifluorescence microscope. Images were captured with an Olympus DP72 camera and analysed with the Olympus CellSens Software package.

### Assessment of Bcl-2 and Bax expression

The expression and cellular location of both Bcl-2 and Bax in the HT-29 and Caco-cell lines were determined by immunofluorescence microscopy. Cells were grown on coverslips (50 000 cells per coverslip), allowed to adhere overnight and exposed to the test nucleosides and camptothecin for 6 hours. After rinsing with PBS, cells were fixed with 3% formaldehyde in PBS for 20 minutes. The cells were rinsed and permeabilized for 5 minutes with 0.25% Triton X100, prepared in PBS with 0.5% bovine serum albumin (BSA). Following permeabilization, cells were blocked with 1% (BSA) in PBS for 1 hour. Thereafter cells were washed and incubated overnight with the respective primary antibodies (Bcl-2 or Bax in PBS with 0.5% BSA) at 4°C. Mouse anti-Bcl-2 and mouse anti-Bax was obtained from Biovision and used at a concentration of 10 mg/mL. After washing with PBS, cells were incubated with species compatible Alexa-fluor 568 (Abcam) secondary antibody. FITC-conjugated phalloidin, (Abcam), was used to visualize the cytoskeleton and the nuclei were visualized with Hoechst 33342 stain as described previously. The cells were viewed using an Olympus BX41 epifluorescence microscope with the appropriate filters for each fluorochrome. Images were captured with an Olympus DP72 camera and analysed with the Olympus CellSens Software package.

### Evaluation of cells for induction of autophagy

Cells were grown on sterile coverslips in 6 well plates 9 (50 000 cells per coverslip) allowed to adhere overnight and treated with 50 μM of test nucleosides. Chloroquine (50 μM) was used as a positive control since it is known to cause extensive autophagic vacuole formation [17, 18]. Cells were exposed to test compounds, chloroquine and camptothecin for 3 hours, washed with PBS and then incubated at 37°C with 50 μM monodansyl-cadaverine (MDC) (Sigma), in PBS for 15 minutes [19, 20, 21]. Camptothecin was included as a negative control for vacuole formation as it failed to cause vacuole formation in the two cell lines. Coverslips were rinsed with PBS and the live cells were viewed under the Olympus BX41 epifluorescence microscope. Images were captured with an Olympus DP72 camera and analysed with the Olympus CellSens Software package.

### Nucleoside test compounds

The novel nucleoside derivatives, nucleoside 1, 2 and 5 evaluated in this study were synthesised in the School of Chemistry, University of the Witwatersrand, and characterized by 1^H^ and ^13^C NMR, IR and HRMS spectroscopy. They were synthesized from a variety of starting materials purchased from Sigma-Aldrich according to modified procedures described in Sproat, 1994 [22]. Nucleoside 2 is a known compound and spectra from the other two compounds are available from the authors. Stock solutions (20 mM) of the test nucleosides were prepared in tissue culture grade DMSO.

### Data analysis and statistics

All the data was analysed and dose response curves constructed using Graph-Pad Prism version 5. Differences between controls and nucleoside treated samples were tested for significance with the Prizm3 Instat software package, using analysis of variance (ANOVA) and the Student’s-t test with a significance threshold set at *p* < 0.05.

## Results

### Nucleoside analogues are cytotoxic to HT-29 and Caco-2 cells

**Fig 1:**
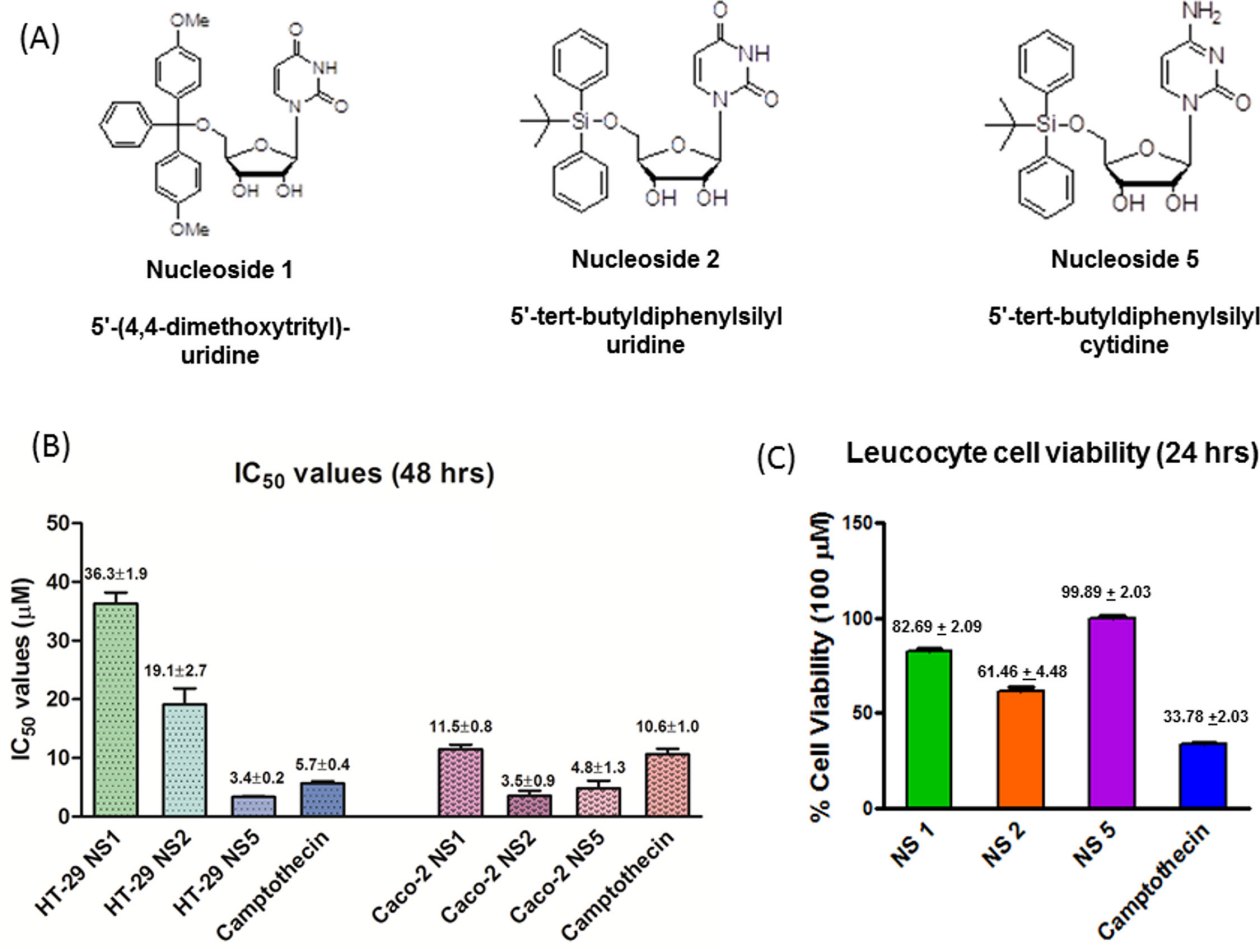
Structures of the active nucleoside analogues (A), their respective IC_50_ values compared to camptothecin, in the Caco-2 and HT-29 cell lines (B), and (C) the leucocyte survival bargraph. (B) The bars represent the mean ± SEM of the IC_50_ values from four MTT assays carried out for each test nucleoside. The HT-29 and Caco-2 cells were exposed to the nucleoside analogues at concentrations ranging from 1 μM to 120 μM. Normal peripheral leukocytes (C) were exposed to 100 μM of nucleoside 1, 2, 5 and camptothecin for 24 hrs.

Three novel pyrimidine ribonucleoside analogues were identified in a screening procedure of twenty three novel nucleosides as having cytotoxic activity against both HT-29 and Caco-2 cell lines when tested at a concentration of 100 μM. The structures of the three active nucleosides are shown in Fig 1A. Nucleoside 1 and 2 were uridine analogues and nucleoside 5 was a cytidine analogue. The pyrimidine bases were linked via an *N*-glycosidic bond to carbon 1 of the ribose sugar. All three molecules were substituted with bulky phenyl groups on carbon 5 of the ribose sugar. Nucleoside 1 had a C-O linkage with a 4,4-dimethoxytrityl group and nucleosides 2 and 5 were linked to a tert-butyldiphenyl group via a stable Si-O bond. The nucleosides were arbitrarily numbered one to twenty-three and test nucleosides number one, two and five were found to be active in the initial screening assays.

The fifty percent inhibitory concentration (IC_50_) values were obtained for the three active nucleosides and are shown in Fig 1B. The two cell lines displayed differential sensitivity to the three active test compounds and to camptothecin. HT-29 cells were more sensitive to camptothecin than Caco-2 cells, with an IC_50_ value of 5.7 μM compared to 10.6 μM, respectively. Nucleoside 5 was the most effective against HT-29 cells with an IC_50_ value of 3.4 μM and showed similar activity to the Caco-2 cells with an IC_50_ value of 4.8 μM. In HT-29 cells, nucleoside 1 and 2 had IC_50_ values 6.4 and 3.4 times higher than that of camptothecin, displaying IC_50_ values of 36.3 and 19.1 μM, respectively. This is in contrast with the Caco-2 cells, where nucleoside 1 had similar activity to camptothecin and nucleoside 2 was 3 times more active than camptothecin, with an IC_50_ value of 3.5 μM. Representative dose response curves are shown in S1 Fig

Exposure of freshly isolated leukocytes to each of the three nucleoside analogues and to camptothecin, respectively indicated that nucleoside 2 had the greatest inhibitory effect on the leukocytes, causing a 38.5% decrease of leucocyte viability at a concentration of 100 μM. This was two-fold lower than the effect of camptothecin on the leukocytes. Nucleoside 1 and nucleoside 5 were the least toxic to the leukocytes, with cell viability being maintained at 82.7% and 99.9%, respectively (see Fig 1C).

### Nucleosides induce caspase 8 and caspase 3 activity

**Fig 2:**
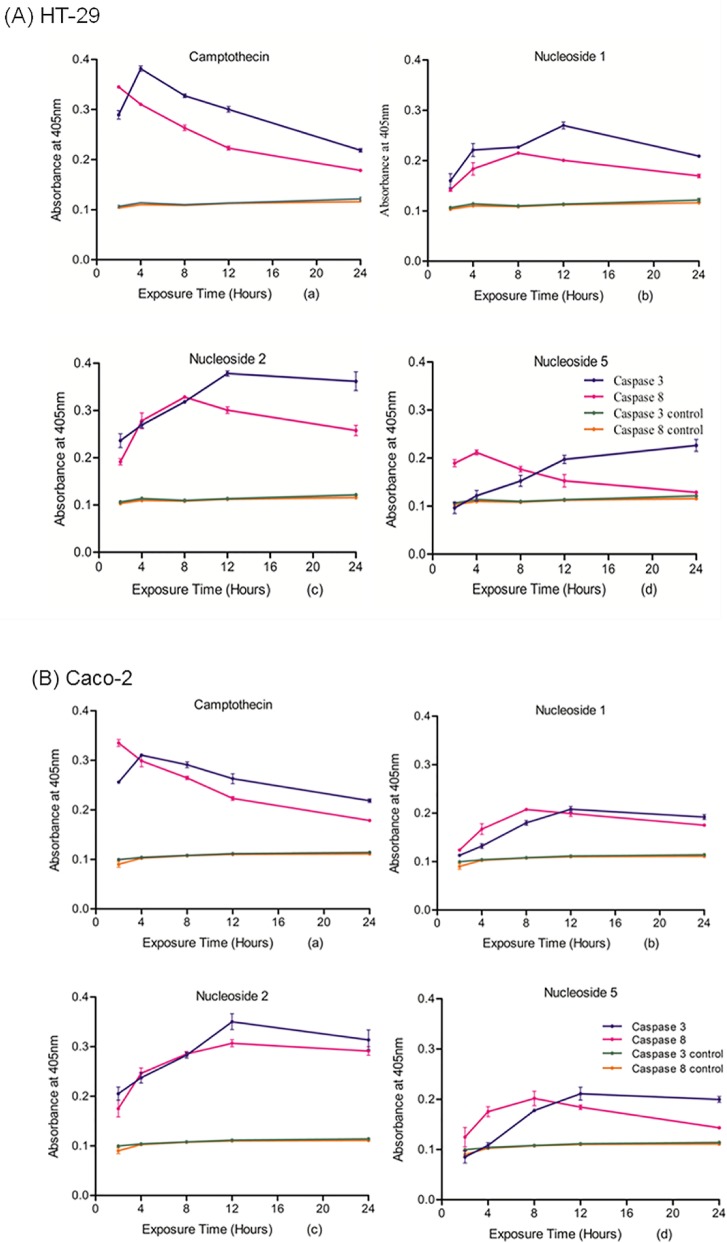
Active nucleosides induce caspase 3 and caspase 8 activity in both HT-29 (A) and in Caco-2 (B) cells after various exposure periods to 100 μM camptothecin (a), nucleoside 1 (b), nucleoside 2 (c), and nucleoside 5 (d) Data points on the graphs represent the mean of the triplicate values of caspase activity after cells were exposed to the nucleoside analogues for the indicated time periods.

Caspase 8 is an initiator caspase which causes cleavage and activation of effector caspases, such as caspase 3. Fig 2 shows caspase 8 and caspase 3 activity determined at various time intervals, following the addition of the test nucleosides, to both cell lines. The three nucleosides caused an increase in caspase 8 and caspase 3 activity in both cell lines relative to the negative control. However, with the exception of nucleoside 2, the caspase activity was not as high as that induced by camptothecin. Nucleoside 2 was the most effective inducer of caspase activity in both cell lines, inducing caspase 8 and 3 activity to a similar level as camptothecin. However, in both the HT-29 and Caco-2 cell lines, the induction of maximal caspase 8 and 3 activity took longer to achieve (12 hours as opposed to 2–4 hours). Importantly, the activity of both caspases declined at a slower rate than that of the camptothecin treated cells, remaining higher than camptothecin after 24 hours of exposure.

### Nucleosides have a minimal effect on mitochondrial membrane potential (Δψm)

The Δψm was measured by flow cytometry with the cationic fluorescent dye JC-1. The percentages of Δψm in the nucleoside treated HT-29 and Caco-2 cells compared with the negative and positive control cells are summarized in Table 1. Compared to the negative control, in the HT-29 cells, nucleoside 2 and 5 had a marginal (< 3%) effect on the Δψm. Nucleoside 1 treatment resulted in 4% of cells with a decreased Δψm above that of the control. In contrast, camptothecin treated cells had 34.8% of cells with a decreased Δψm compared to the control. In the Caco-2 cells, nucleosides 1 and 5 affected some 17% of cells resulting in a decreased Δψm, while nucleoside 2 had a marginal effect (< 3%) above that of the control. This is in contrast with camptothecin which caused 34.6% cells with a Δψm in the Caco-2 cells. Scattergraphs are shown in supplementary S2 Fig.

**Table 1:** Summary of the mitochondrial membrane potential change induced by the nucleoside analogues and camptothecin in the HT-29 and Caco-2 colon cancer cell lines.

	Percentage cells with unchanged Δψm		Percentage cells with depolarized Δψm	
Cell type	HT-29	Caco-2	HT-29	Caco-2
**Untreated cells**	88.44	92.58	10.35	7.47
**Nucleoside 1**	79.55	75.64	14.39	24.33
**Nucleoside 2**	87.02	89.46	12.74	10.38
**Nucleoside 5**	87.53	75.68	12.12	23.96
**Camptothecin**	53.59	57.96	44.00	40.88

The table shows the percentage of HT-29 and Caco-2 cells with a depolarized ΔΨm and an unchanged ΔΨm after exposure to the nucleoside analogues at a concentration of 100 μM for 24 hours.

### Nucleosides alter intracellular cytochrome c distribution

**Fig 3:**
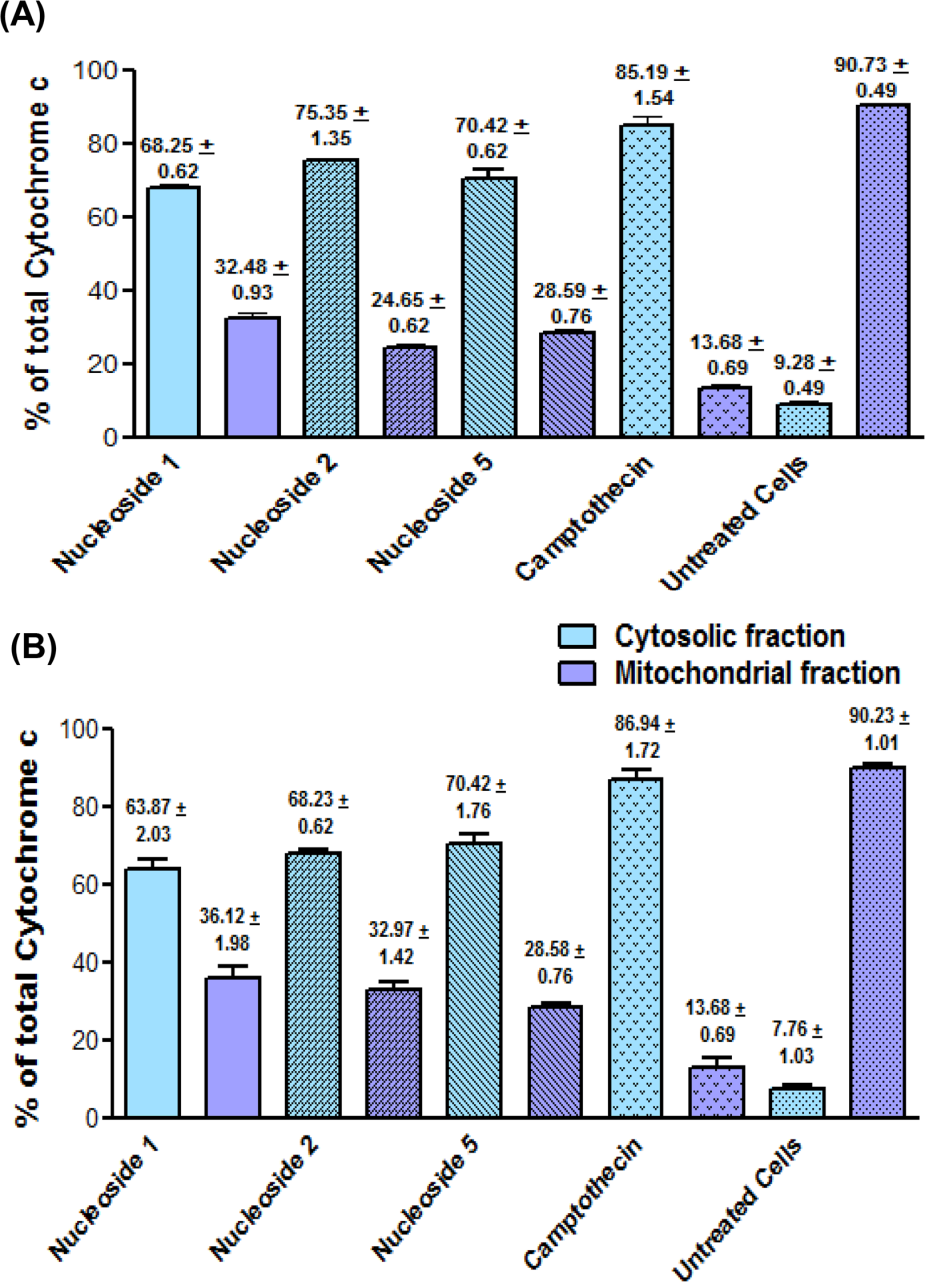
Nucleosides 1, 2 and 5 cause the redistribution of cytochrome c from mitochondria to the cytosol in HT-29 (A) and Caco-2 (B). The cells were exposed to 100 μM of nucleoside 1, 2, 5 and camptothecin, respectively, for 24 hours. The bars represent the mean ± SEM from three Elisa assays carried out for each nucleoside.

In cells, between 90 and 100% of total cellular cytochrome c is normally located within the mitochondria. Fig 3A and 3B show that the test nucleosides increased the cytosolic cytochrome c fraction for both cell lines relative to the negative control, but not to the same extent as camptothecin. In the HT-29 cells, nucleoside 2 caused the highest percentage of cytochrome c to relocate to the cytosol (75.35±1.15%), albeit lower than that of camptothecin (85.19±1.54%). Nucleosides 1 and 5 caused some 68.22±0.62% and 70.42±0.62% of cytochrome c to relocate to the cytosol, respectively.

In the Caco-2 cells, nucleoside 5 caused the highest percentage of cytochrome c to shift to the cytosol (70.42±1.76%), which is lower than that of camptothecin (86.94±1.72%). The effects of nucleosides 1 and 2 were comparable to nucleoside 5 with cytosolic cytochrome c fractions of 63.87±2.03% and 68.23±0.62%, respectively. The results thus indicate that treatment with the nucleoside analogues caused a decrease in mitochondrial cytochrome c and concomitant increase in cytosolic cytochrome c.

### Annexin V binding increases in HT-29 and Caco-2 cells

The flow cytometric assay for annexin V-FITC, indicates that apoptosis was induced after addition of the active nucleoside analogues to both cell lines, as shown in Table 2 and S3 Fig. After 24 hours of exposure to the nucleoside analogues or camptothecin, there were primarily two populations of cells: viable, non-apoptosing cells (annexin V-FITC and PI negative) and cells undergoing apoptosis (annexin V-FITC positive and PI negative). A small population of cells were observed to be annexin V-FITC and PI positive, indicating that they were in end-stage apoptosis or necrosis.

**Table 2:** Percentage of HT-29 and Caco-2 cells in early and late apoptosis as determined by the annexin-V assay.

	Percentage viable cells		Percentage early apoptotic cells		Percentage late apoptotic / necrotic cells	
	HT-29	Caco-2	HT-29	Caco-2	HT-29	Caco-2
**Untreated control**	86.47	79.06	4.28	7.34	3.22	0.18
**Nucleoside 1**	55.08	61.38	38.41	17.42	5.94	14.84
**Nucleoside 2**	68.39	66.34	20.52	21.68	10.58	11.41
**Nucleoside 5**	74.53	62.59	22.26	30.95	2.33	4.83
**Camptothecin**	47.95	50.18	27	27.06	19.64	17.78

The percentage of each subpopulation of cells in both cell lines exposed to the nucleoside analogues is shown in Table 2. In the HT-29 cells, nucleoside 1 was the most effective apoptotic inducer, with 38.4% of the cells being detected in early apoptosis and 5.9% of cells in late apoptosis/necrosis. While superior to camptothecin for early apoptosis, camptothecin treatment nevertheless caused some 19.6% of cells to shift to late apoptosis/necrosis. This indicates that camptothecin was able to induce apoptosis at a faster rate than the nucleosides. Nucleoside 2 treatment resulted in 20.5% of cells being in early apoptosis and 10.6% in late apoptosis. Nucleoside 5 had 22.2% cells in early apoptosis and 2.3% cells in late apoptosis/necrosis.

The effects were different in the Caco-2 cells, all three nucleosides showed a similar percentage of viable cells, ranging from 61 to 66.3%. Nucleoside 5 was the most effective with 30.9% of cells being detected in early apoptosis and 4.8% in late apoptosis or necrosis, whereas after nucleoside 1 treatment, 17.4% of cells were in early apoptosis and 14.8% in late apoptosis. Some 21.7% cells were detected in early apoptosis and 11.4% of cells in late apoptosis/necrosis following nucleoside 2 treatment. Scattergraphs are shown in S3 Fig.

### The nucleosides fail to induce caspase 9 activity

The ability of the test nucleosides to stimulate caspase 9 activity was determined after cells were exposed to 50 μM of each nucleoside for 24 hrs. Camptothecin (20 μM) was used as a positive control and was able to stimulate caspase 9 activity, in contrast with the three nucleosides as shown in S4 Fig.

### Nucleosides do not affect the expression of Bcl-2 and Bax

In order to gain more information regarding the redistribution of cytochrome c to the cytoplasm without a prominent change in mitochondrial membrane potential, we investigated the expression of Bcl-2 and Bax in the HT-29 and Caco-2 cell lines and their response to nucleoside treatment. Cells were exposed to 50 μM nucleosides for 6 hours.

#### Bcl-2 is strongly expressed and associated with the nucleus in both HT-29 and Caco-2 cells

In untreated and treated HT-29 cells, Bcl-2 was expressed with a strong fluorescent signal and associated with the nucleus (S5 Fig). This was confirmed by merging Hoechst and Bcl-2 images which presented a pink merged image indicating co-localisation of the two fluorochromes. Exposure to the test nucleosides had no obvious effect on Bcl-2 expression levels or its subcellular distribution. Caco-2 cells similarly, had a predominantly nuclear distribution of Bcl-2. However some cells emitted a much weaker signal than others (S6 Fig). Exposure to nucleoside 1 and 2 caused minimal redistribution of Bcl-2 to the perinuclear space which was not observed with nucleoside 5 treatment.

#### Bax is poorly expressed in HT-29 and Caco-2 cells with a perinuclear distribution

In HT-29 control cells, Bax was sparsely associated with the nuclei with a predominantly perinuclear distribution (S7 Fig). Hoechst/Bax merged images support the observation that Bax has a perinuclear distribution. Noteably, the fluorescent intensity of Bax was much lower than that of Bcl-2 indicating comparatively lower expression levels. Exposure to the three nucleosides showed a small increase in Bax fluorescence compared to that of the control. In addition, treatment with nucleoside 5, caused a polarised nuclear clustering of Bax (S7 Fig). The Hoechst/Bax merged image shows an increase in pink fluorescence indicating an increased association of Bax with the nucleus.

Expression and cellular distribution of Bax was similar in Caco-2 cells (S8 Fig). Bax expression was low in Caco-2 cells and images had to be enhanced to show the subcellular distribution. It was therefore not possible to determine if the nucleosides had a direct effect on Bax expression levels or its subcellular distribution in Caco-2 cells.

### Effects of the nucleosides on cell morphology

#### Hoechst staining identifies apoptotic nuclei

**Fig 4:**
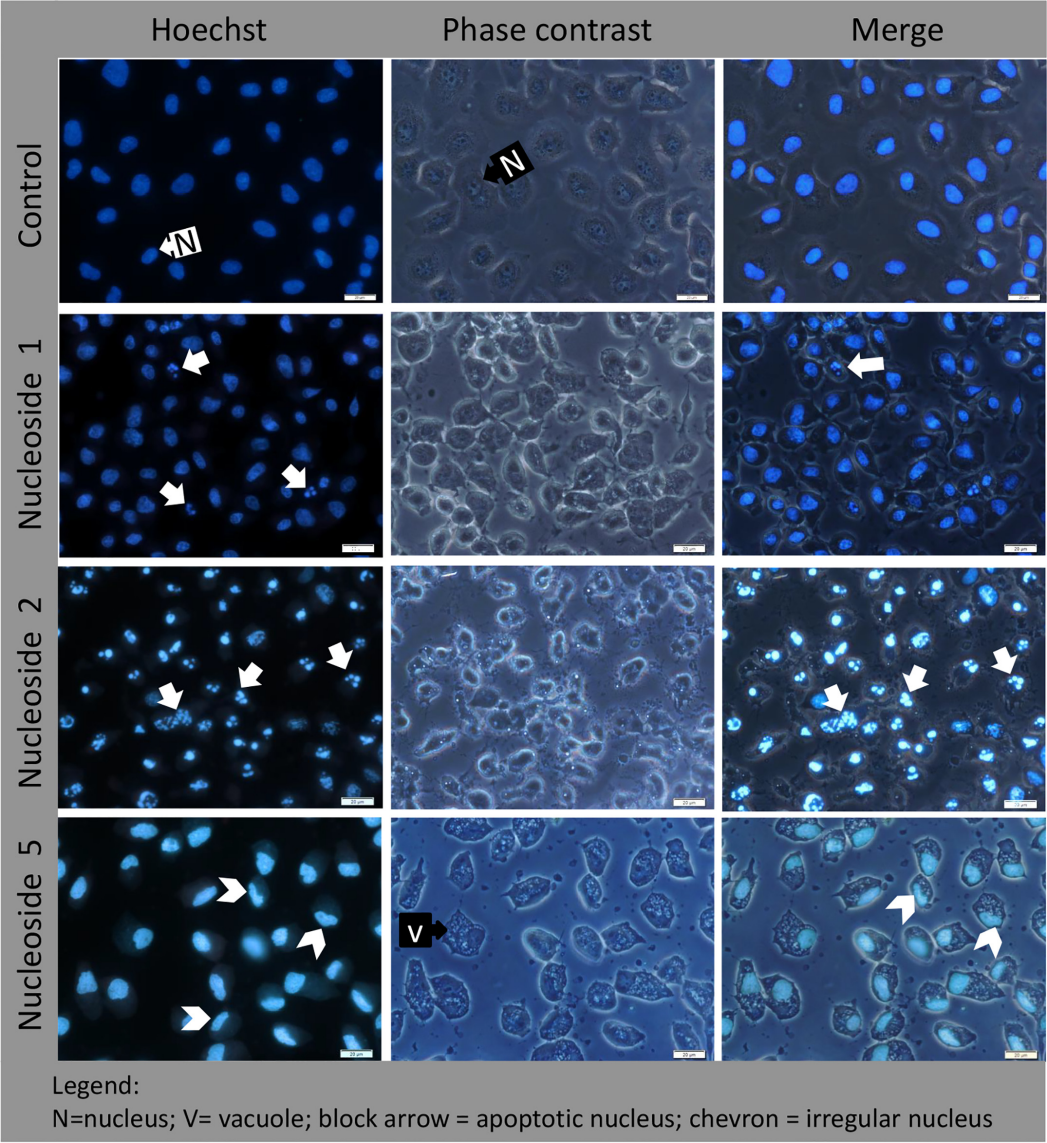
In HT-29 cells, nucleoside 1 and 2 induce nuclear fragmentation, while nucleoside 5 causes a loss of normal nuclear structure and cytoplasmic vacuolisation. Cells were exposed to 50 μM of nucleoside 1, 2 and 5 for 24 hours and stained with Hoechst 33342 stain. Scalebar: 20 μm.

In HT-29 cells, both nucleoside 1 and 2 were able to induce apoptosis as reflected by the specific nuclear staining pattern (indicated with a solid/block arrow in Fig 4). The nuclei of untreated control cells were typically oval in shape, staining blue with a uniform distribution of the dye. In contrast, the nucleoside 5 treated cells that remained attached to the growth surface after 20 hours, demonstrated irregularly shaped nuclei (Fig 4), indicating that the cell nucleus may be a possible target. In addition, these cells showed a high degree cytoplasmic vacuolisation (Fig 4), indicating an extensive cytoplasmic effect.

**Fig 5:**
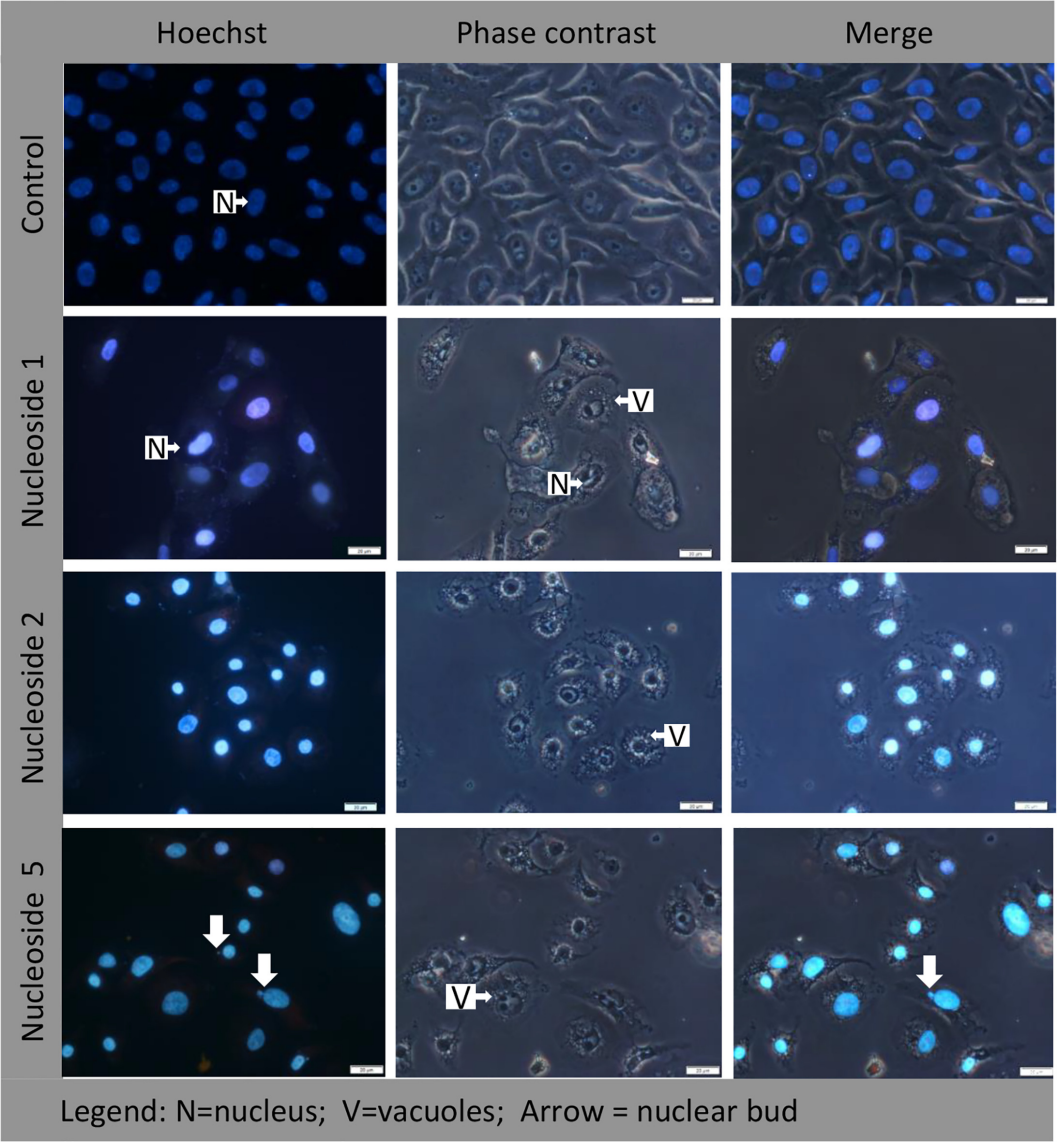
In Caco-2 cells, nucleoside 1 and 2 cause perinuclear vacuole formation, while nucleoside 5 causes a loss of normal nuclear structure, nuclear budding and cytoplasmic vacuolisation. Cells were exposed to 50 μM of nucleoside 1, 2 and 5 for 24 hours and stained with Hoechst 33342 stain. Scalebar: 20 μm.

Untreated Caco-2 cells showed the typical oval nuclear structure with even dye distribution when stained with Hoechst (Fig 5). Staining of treated Caco-2 cells did not demonstrate a distinct apoptotic nuclear staining pattern as seen for nucleoside 1 and 2 in HT-29 cells. However, those cells that remained attached to the growth surface contained contracted nuclei with increased fluorescence intensity, indicating a pre-apoptotic state. Perinuclear vacuole formation was a prominent feature of both nucleoside 1 and 2. While nucleoside 1 did not have an obvious effect on the nuclei of treated cells, the nuclei of cells treated with nucleoside 2 appeared to be contracted. The increased fluorescence intensity observed for these nuclei is indicative of a pre-apoptotic state. In contrast, the nuclei of cells exposed to nucleoside 5 showed variation in the sizes of the nuclei, irregular staining of the nuclei and the presence of small nuclear buds as indicated by white arrows in Fig 5.

#### Nucleoside 1 and 2 cause rapid vacuole formation

**Fig 6:**
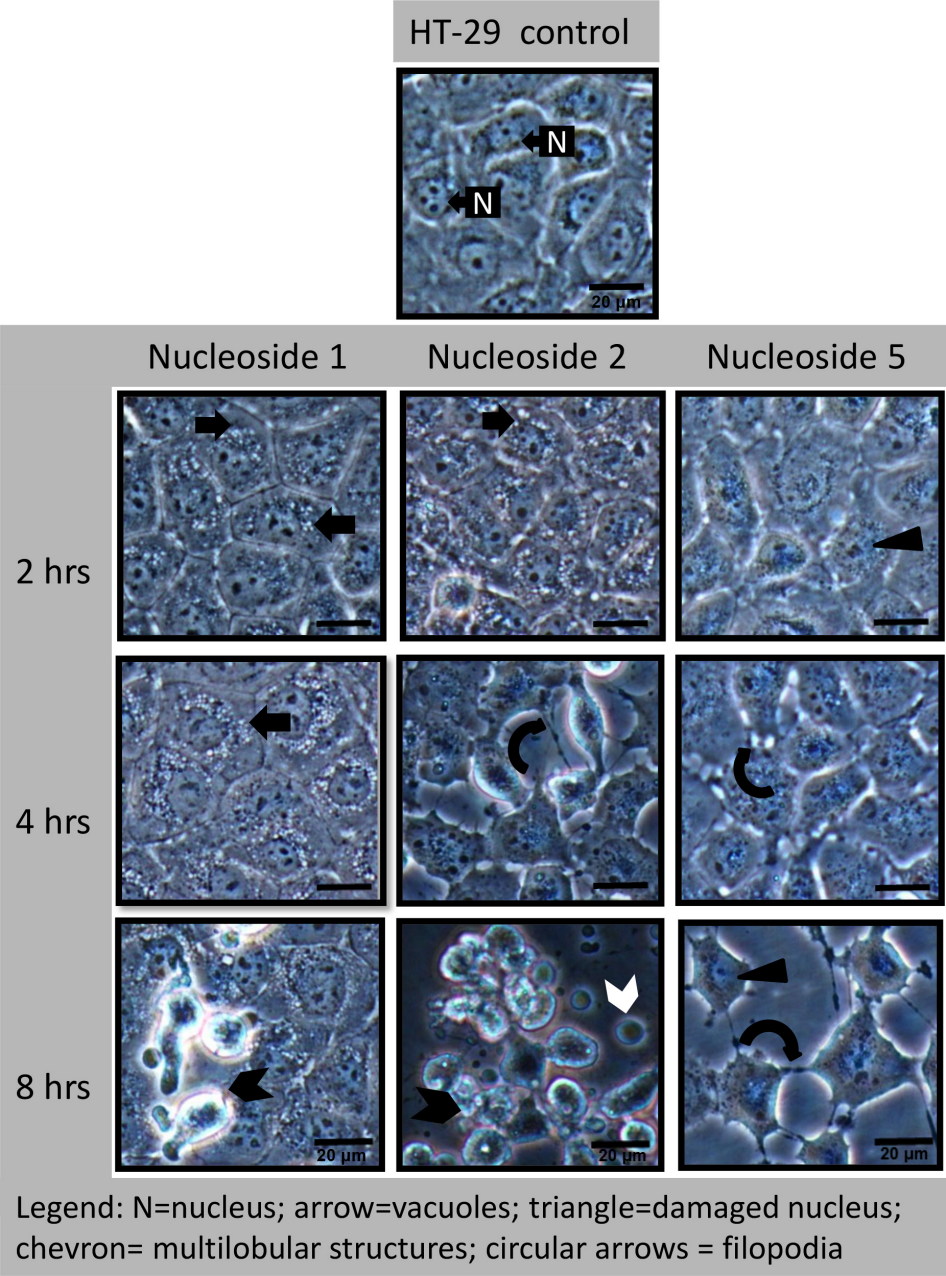
Nucleoside-induced morphological changes in HT-29 cells include rapid perinuclear vacuole formation (nucleoside 1 and 2) and a loss of cellular adherence (nucleoside 5). Cells were exposed to 50 μM of the nucleoside for the indicated time periods. Experiments were repeated three times. Scale bar: 20 μm.

In the HT-29 cells, nucleoside 1 and 2 caused the appearance of perinuclear vacuoles as soon as two hours post-exposure (solid/block arrows in Fig 6). At this time, the nucleus and nuclear structure appeared to be intact. At four hours, nucleoside 1 caused an increase in size and number of the vacuoles, while a loss of cell adherence to the growth surface was a prominent effect of nucleoside 2, as indicated by the curved arrow (Fig 6). At six to eight hours, nucleoside 1 caused some cells to detach from the culture surface and form an unusual multi-lobular structure (chevron in Fig 6). Cells that remained attached showed a loss of nuclear definition and a change in the nuclear shape together with a loss in the resolution of the nucleoli. A similar trend was observed for nucleoside 2, except that the process was accelerated with a large percentage of cells detaching from the growth surface from 4 hours onwards (curved arrow in Fig 6). The cells appeared rounded at eight hours with numerous multi-lobular cells and smaller rounded structures (white chevron in Fig 6) being present.

**Fig 7:**
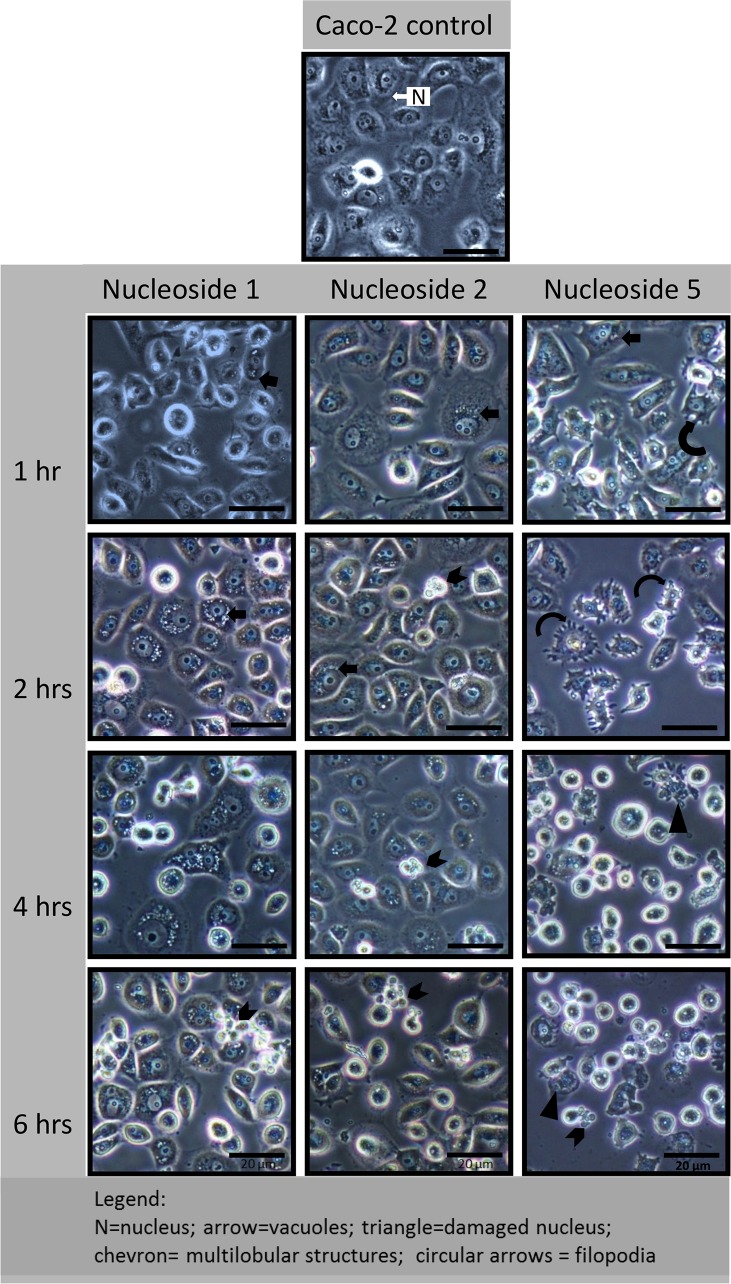
Nucleoside-induced morphological changes in Caco-2 cells include rapid perinuclear vacuole formation (nucleoside 1 and 2) and a loss of cellular adherence (nucleoside 5). Cells were exposed to 50 μM of each nucleoside for the indicated times and the experiments were repeated three times. Scalebar: 20 μm.

In Caco-2 cells, the morphological changes were similar to that of HT-29 cells with the exception being the rate at which the various changes occurred. Perinuclear vacuole formation was observed within 1 hour of exposure (Fig 7) and was a prominent feature of Caco-2 cells exposed to nucleoside 1 and 2 (Fig 7). The appearance of the multi-lobular cell structures (indicated by a chevron) was evident from two hours onwards for nucleoside 2 and from 4 hours onwards for nucleoside 1 and 5. As seen in Fig 7, in Caco-2 cells, nucleoside 2 treatment did result in the loss of cell adherence to the growth surface, as it did in HT-29 cells.

**Fig 8:**
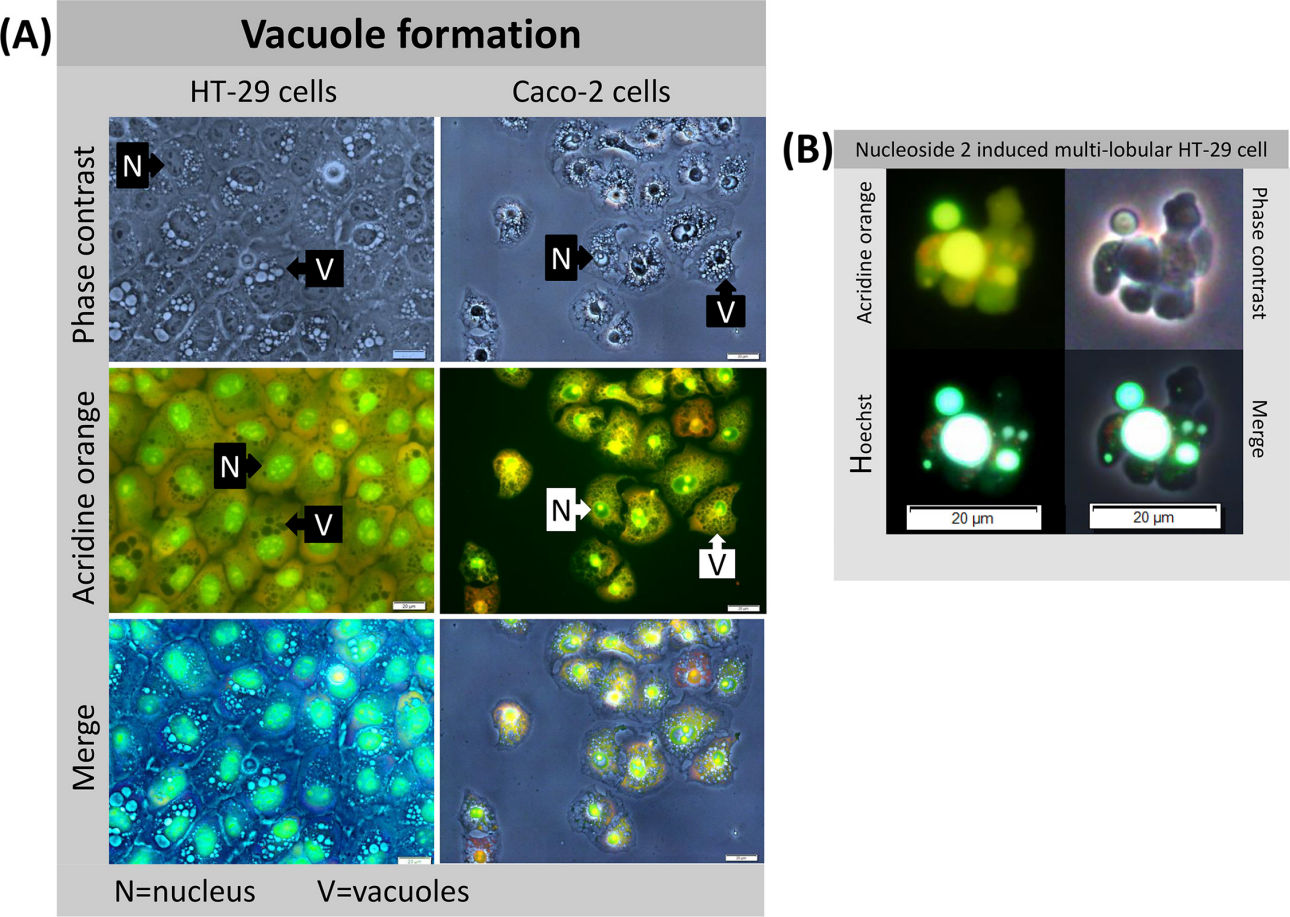
Nucleoside 2 treated vacuolated HT-29 and Caco-2 cells stained with acridine orange shows an absence of lysosomal staining (A) and a nucleoside 2 induced HT-29 multi-lobular structure (B). Acridine orange stained HT-29 and Caco-2 cells, following exposure to 50 μM of nucleoside 2 (A). HT-29 cells were exposed to 50 μM of nucleoside 1 and the supernatant harvested and centrifuged at 500 x *g* for 5 mins. The multi-lobular cell was stained with Hoechst 33342 and acridine orange (B). Scalebar: 20 μm.

In cell culture, apoptotic cells detach rapidly from the growth surface and disintegrate in the culture medium. It is therefore not always possible to observe a high apoptotic index (cells with fragmented nuclei) in *in vitro* cultures. However, Hoechst staining of detached Caco-2 cells indicate that a high percentage of cells were apoptotic. Notably, multi-lobular cells were frequently observed in the nucleoside treated cultures of both Caco-2 and HT-29 cells, and may well represent an apoptotic cell prior to disintegration (Fig 8B). The formation of these structures, while more plentiful in the treated Caco-2 cells, was also observed in the HT-29 cells.

#### Nucleoside 5 damages intracellular junctions

In contrast, HT-29 cells exposed to nucleoside 5, showed a rapid change in the integrity of the intra-cellular junctions and a rapid loss of nuclear definition (solid triangle, Fig 6). The formation of filopodia was evident at two hours. At eight hours, a large percentage of cells remained loosely attached to the growth surface by extended filopodia (rounded arrows) and showed a distinct loss of normal cellular morphology. Although vacuole formation was not obvious in the cells exposed to nucleoside 5 for six hours, an exposure period of 24 hours revealed extensive vacuole formation in the cells remaining attached to the growth surface, shown in Fig 8A. Hoechst and acridine orange staining revealed that the multi-lobular cells had fragmented nuclei and may well present a late apoptotic cell (Fig 8B). These structures were frail and rapidly disintegrated in the cell culture media.

Caco-2 cells responded in a similar fashion to nucleoside 5 which caused a rapid change in the shape of the cells, with the loss of cellular junctions (rounded arrow, Fig 7), 1 hour post treatment. This alteration in cell-cell contacts progresses rapidly, with a high percentage of cells being non-adherent after six hours (Fig 7). The remaining adherent cells display altered morphology with both nuclear and cytoplasmic changes (solid triangles, Fig 7).

#### Nucleoside 5 causes actin aggregation

**Fig 9:**
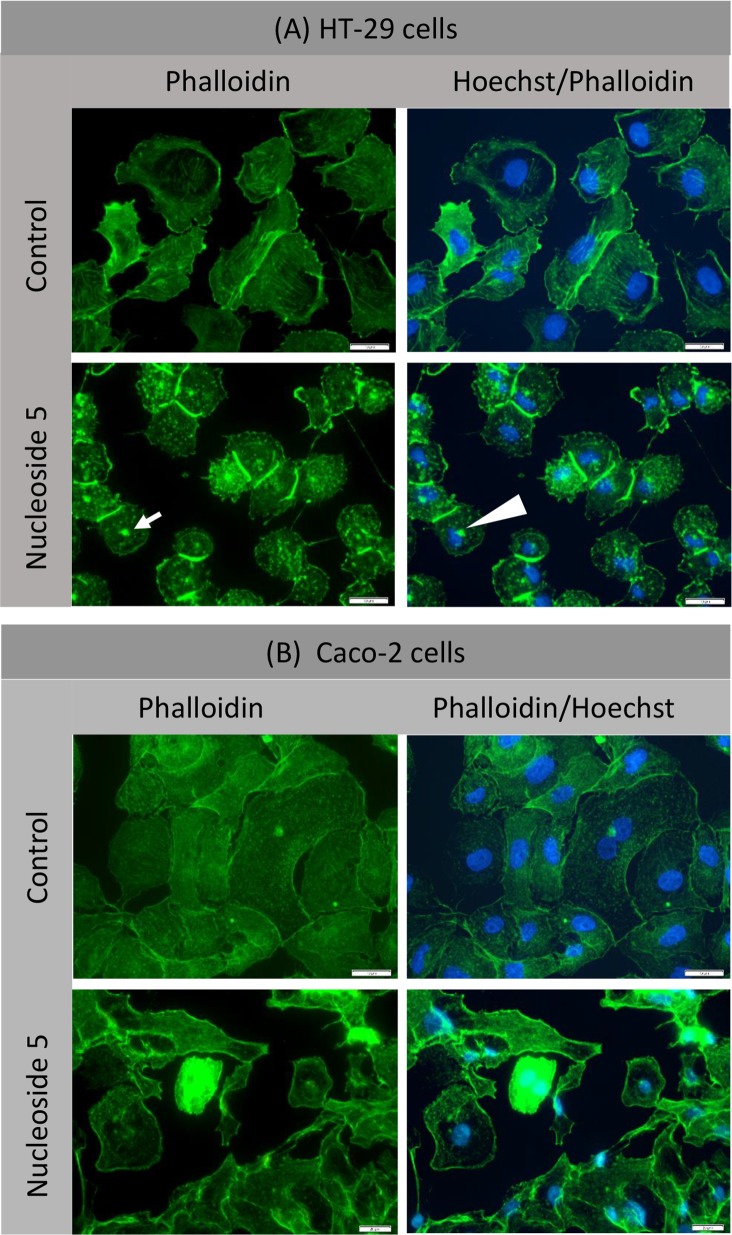
Nucleoside 5 causes aggregation of actin in HT-29 cells, but not in Caco-2 cells. Cells were treated with 50 μM of nucleoside 5 for 6 hours. Scalebar: 20μm.

Since phalloidin binds to F-actin and may provide clues regarding cytoskeletal changes induced by the nucleosides, nucleoside treated cells were incubated with a phalloidin-FITC conjugate. Nucleoside 5 induced a marked change in the subcellular distribution of actin in HT-29 cells but not in Caco-2 cells (Fig 9). In HT-29 cells actin aggregated in clusters (arrow in Fig 9) which were distributed throughout the cytoplasm and in some cells was associated with the distorted nucleus (solid triangle in Fig 9). Despite the fact that the cytoskeleton was affected, there was an aggregation of actin at the remaining intercellular junctions (Fig 9). Nucleoside 1 and 2 treatment did not cause any changes in actin distribution in either HT-29 or Caco-2 cells.

### Vacuoles and autophagy

#### Vacuoles lack autophagic features

**Fig 10:**
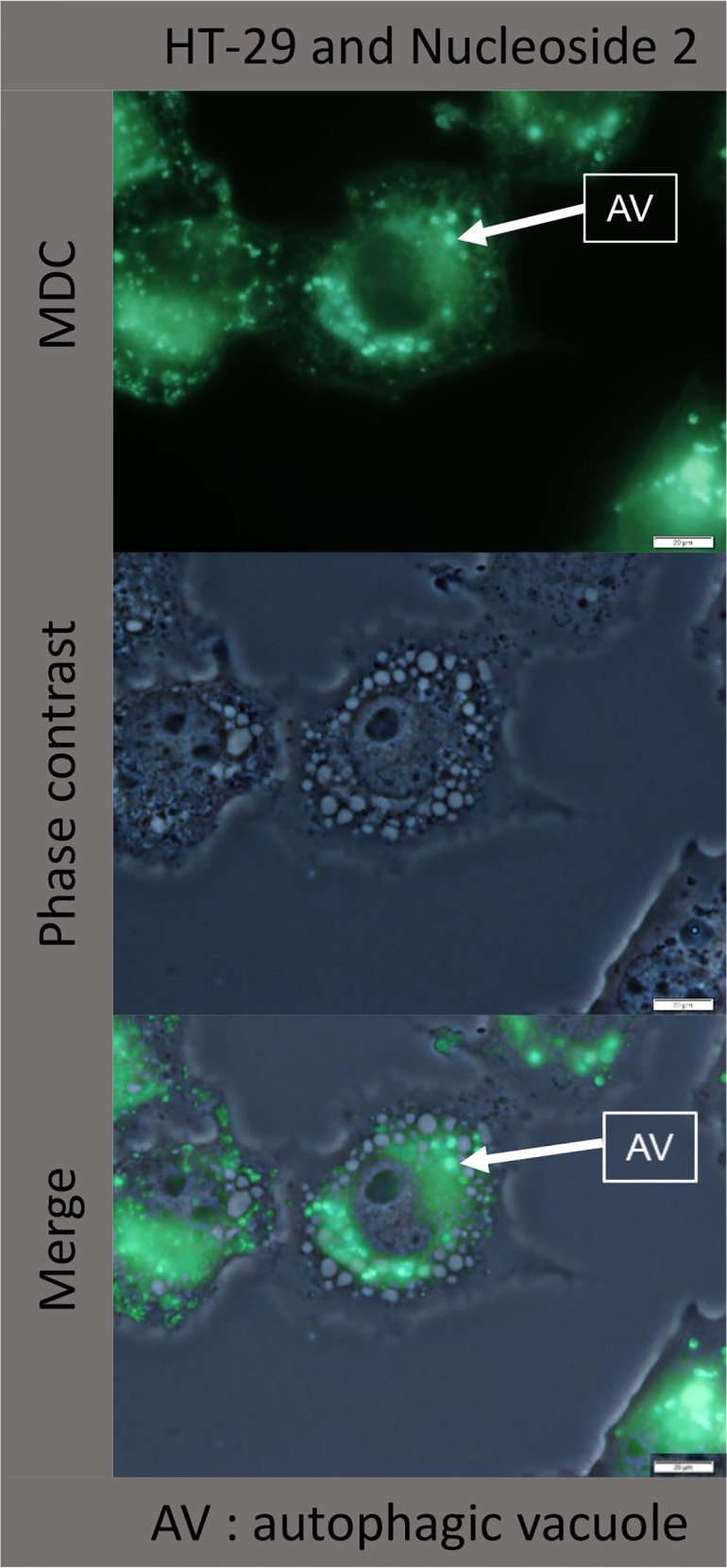
Vacuoles observed under phase contrast microscopy do not co-localize with MDC stained fluorescent granules. HT-29 cells were exposed to 50 μM of nucleoside 2 for 3 hours before MDC staining.

In order to determine the nature of the vacuoles observed under phase contrast microscopy, nucleoside 2 treated cells were first stained with acridine orange, an indicator of acidic structures, including lysosomes and autophagosomes. Since no organelles were stained bright orange/red with acridine orange, (Fig 8A), it is unlikely that such vacuoles were autophagic in nature. The non-autophagic nature of these vacuoles was subsequently confirmed by staining nucleoside treated cells with monodansylcadaverine (Fig 10). Analysis of phase contrast and MDC labelled merged images, revealed that these vacuoles do not co-localize with the MDC granules, thus indicating their non-autophagic nature (Fig 10). Moreover, the vacuoles appeared to be fluid-filled and not surrounded by membranes filled with cytoplasm, as would be expected for autophagic vacuoles.

#### Nucleoside 2 and nucleoside 5 induce the formation of autophagic vacuoles

**Fig 11:**
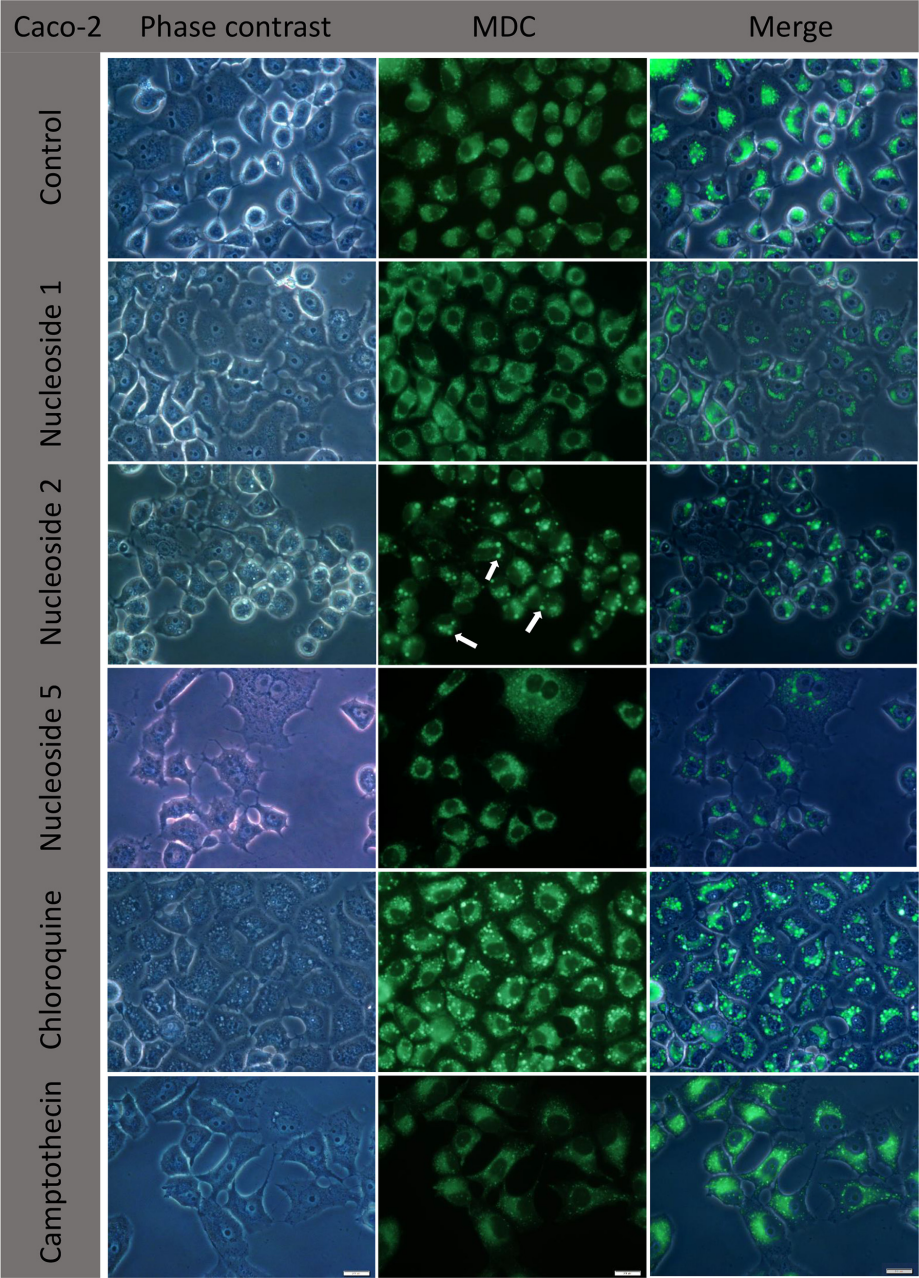
Nucleoside 2 and chloroquine cause the formation of large, highly fluorescent autophagic vacuoles in Caco-2 cells. Cells were exposed to nucleoside 1, 2 and 5 at a concentration of 50 μM for three hours. Chloroquine (50 μM) was used as a positive control and camptothecin (20 μM) was included as a vacuole negative control. Arrows indicate autophagic vacuoles. Scalebars: 20 μm.

The question regarding autophagy was further investigated using MDC, an indicator for autophagic vacuoles. Chloroquine was used as a positive control. In Caco-2 cells, both chloroquine and nucleoside 2 showed a similar pattern of MDC fluorescence of round larger, and brightly fluorescent granules with a perinuclear distribution (arrows, Fig 11). The diameter of these granules varied between 2 and 5 μm as measured by the CellSense software. Small punctate fluorescent granules (less than 1 μm in diameter) and diffuse fluorescence was also present in the cytoplasm surrounding the nucleus. In the control, nucleoside 1 and camptothecin treated cells, small punctate fluorescent granules (<1μm) and diffuse fluorescence showed a perinuclear distribution. Nucleoside 5 caused the formation of larger fluorescent granules, similar in size to the chloroquine induced granules, but with a decreased fluorescence intensity. These granules co-localized with the dark granules visualized under phase contrast.

Analysis of the merged images revealed that the larger, highly fluorescent granules of the chloroquine and nucleoside 2 treated cells do not always co-localize with the vacuoles seen under phase contrast (Fig 10). In the case of nucleoside 2, the bright fluorescent granules co-localized with the prominent phase contrast observed vacuoles. Closer inspection of phase contrast images show that these vacuoles appear to be surrounded by membranes, unlike the vacuoles that do not co-localize with the fluorescent granules. In chloroquine treated cells there was poor co-localisation of phase contrast visualised vacuoles and fluorescent granules (Fig 11).

**Fig 12:**
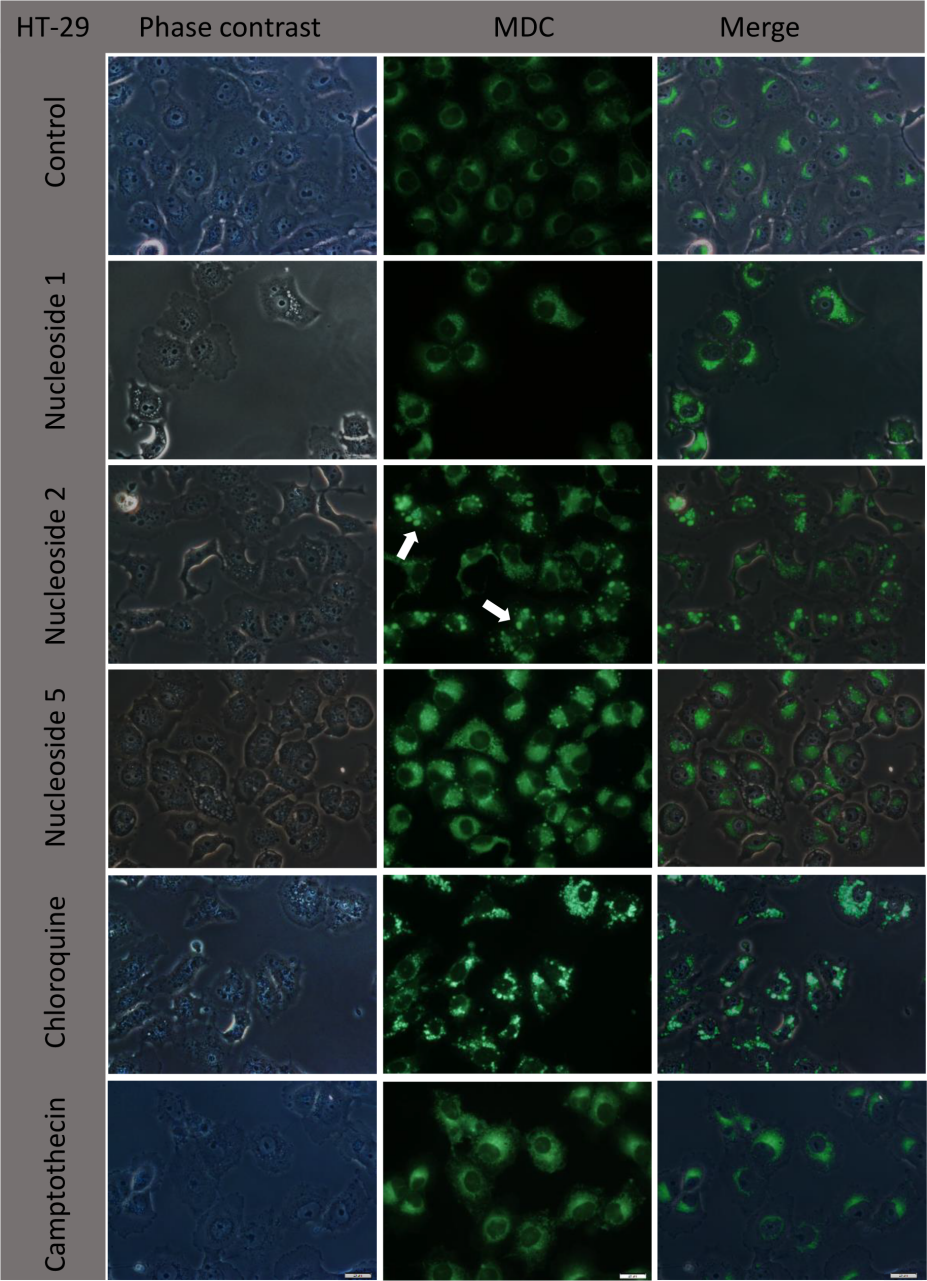
Nucleoside 2 and chloroquine cause the formation of large highly fluorescent autophagic vacuoles in HT-29 cells. Cells were exposed to nucleoside 1, 2 and 5 at a concentration of 50 μM for three hours. Chloroquine (50 μM) was used as a positive control at and camptothecin (20 μM) was included as a negative control. Arrows indicate autophagic vacuoles. Scalebars: 20 μm.

A similar pattern was observed when HT-29 cells were treated with the nucleosides, chloroquine and camptothecin, (Fig 12). Control cells display diffuse and small punctate perinuclear fluorescent granules less than 1 μm in diameter. Nucleoside 1 was similar to the control, but with an increased fluorescence intensity, especially in cells where numerous small vacuoles were visualized with phase contrast microscopy. Nucleoside 2 and chloroquine treated cells displayed a similar increase in larger (2–5 μm diameter) intensely fluorescing granules, as well as smaller punctate fluorescent granules concomitant with a decrease in the diffuse cytoplasmic fluorescence. In HT-29 cells the large fluorescent granules and dark granules observed under phase contrast co-localized when exposed to nucleoside 2 (Fig 12). In cells showing this increased granular fluorescence the diffuse fluorescence was decreased and fewer punctate fluorescent granules were present. Small punctate fluorescent granules that concentrated around the nucleus were observed in the camptothecin treated cells. Nucleoside 5 treated HT-29 cells had a similar staining pattern to Caco-2 cells with larger granules showing less intense fluorescence compared to chloroquine and nucleoside 2 treatment (Fig 12).

## Discussion

Nucleoside analogues are an important class of chemotherapeutic agents used in the treatment of cancer and viral infections [23, 24, 25]. This study identified novel nucleoside analogues that proved to be cytotoxic and able to induce an unusual apoptotic response with the concurrent formation of cytoplasmic vacuoles in two colorectal adenocarcinoma cell lines. Importantly, they demonstrated a sparing effect on normal leukocytes which may translate to a clinically beneficial outcome. Camptothecin, a mammalian topoisomerase I inhibitor, was used as a positive control and is a known inducer of apoptosis, mediating its activity via both the death receptor and the mitochondrial pathway [26, 27].

In Caco-2 cells, the compounds displayed similar or better cytotoxic effects when compared to camptothecin. Only nucleoside 5 displayed superior activity to camptothecin in HT-29 cells. However, all the compounds were comparable to camptothecin in their ability to induce apoptosis. Importantly, at a concentration of 100 μM, they were considerably less toxic than camptothecin to normal leukocytes, which indicates some degree of selectivity towards cancer cells. Nucleosides 1 and 5 showed the least toxicity towards leukocytes, with the percentage cell viability being 82 and 99%, respectively. Although nucleoside 2 was the most toxic decreasing the viability to 60%, it was nevertheless lower than camptothecin, which reduced leucocyte viability to 34%. The decreased toxicity to leukocytes is a positive indicator for cancer cell selective toxicity, since most nucleoside based chemotherapeutic agents are toxic to leukocytes and an indicator of an unfavourable adverse effect profile.

The structures of the three nucleosides (Fig 1A) are such that they cannot undergo phosphorylation to the active triphosphate form, which is a substrate requirement for binding to DNA and RNA polymerase and for the subsequent incorporation into DNA or RNA strands, or the inhibition of the respective polymerase enzymes. It is however possible, that nucleoside one and two may have compromised the activity of other enzymes required in nucleoside processing, such as thymidylate synthase and ribonucleotide reductase. Since nucleoside 1 and 2 have a uridine base they may bind and interfere with the action of thymidylate synthase which is required for the conversion of uridine to thymidine. All three compounds can potentially interfere with the action of ribonucleotide reductase which is responsible for converting ribonucleotides to deoxyribonucleotides [28]. Compromised activity of these two enzymes may explain the cytotoxic and apoptotic effects observed in this study.

Activation of caspase provides evidence of the induction of apoptosis [29, 30]. The impaired expression and function of caspase 8 promotes tumour formation, progression and treatment resistance in several types of cancers [29]. Caspase 3, the key executioner of apoptosis, is responsible for the proteolysis of proteins essential for DNA repair such as caspase activated DNase (CAD) and poly-(ADP-ribose) polymerase (PARP). Upon activation of the extrinsic pathway of apoptosis, it is expected that caspase 8 activation occurs before the activation of caspase 3 [29, 31].

This study showed that the sequential activation of caspase 8 and caspase 3 was induced by all three nucleosides and camptothecin, in both the HT-29 and Caco-2 colorectal cell lines. Since both caspase 8 and caspase 3 were activated, this suggests the involvement of the death receptor pathway. Nucleoside 2 was the most potent compound and induced caspase 3 activity in HT-29 and Caco-2 cells to a similar level as camptothecin, albeit at a slower rate (8 hours *versus* 2 hours for caspase 8 activity and 12 hours *versus* 4 hours for caspase 3 activity). Furthermore, in both cell lines, the increase in caspase 8 and caspase 3 activity was sustained for a longer period of time compared to that of camptothecin. Although nucleoside 2 showed higher caspase activity relative to the other nucleosides, it displayed a lower cytotoxic effect than camptothecin on the HT-29 cell line, with an associated IC_50_ value of 19.1±2.7 μM; but with a potent IC_50_ value of 3.5±0.9 μM in the Caco-2 cell line. The higher IC_50_ values observed for HT-29 cells while not consistent with the observed apoptotic effects, may have been as a result of a slower onset of mitochondrial toxicity. The MTT assay measures the integrity of mitochondrial function and it is possible that mitochondrial dysfunction manifested later than the measured apoptotic markers [32]. Fluorescence microscopy using Hoechst DNA stain showed that the HT-29 cells displayed a high apoptotic index when exposed to nucleoside 2. This is consistent with the increased in caspase 8 and 3 activity. Compared to the control, nucleosides 1 and 5 induced caspase 8 and 3 activation in both cell lines, albeit lower than camptothecin and nucleoside 2, indicating their ability to induce apoptosis. Apoptotic nuclei were readily observed in HT-29 cells treated with nucleoside 1 and 2.

The annexin-V assay, being a measure of the percentage cells that have phosphatidylserine exposed on the outer leaflet of the cell membrane, provides further evidence that the test nucleosides were able to induce apoptosis in both the HT-29 and Caco-2 cells. Externalization of phosphatidylserine occurs in early apoptosis [33]. Although nucleoside 2 showed maximal caspase activity for both cell lines, fewer cells stained for early and late apoptosis when compared to camptothecin. Nucleoside 1 was the most effective in HT-29 cells, whereas nucleoside 5 was the most effective in Caco-2 cells. This indicates some selectivity in terms of phosphatidylserine translocation. Camptothecin treated cells showed the highest percentage of cells in late apoptosis/necrosis indicating its ability to induce apoptosis rapidly. The apparent slower induction of phosphatidylserine translocation in the presence of test nucleosides may be attributed to the intrinsic dysregulation and abnormalities of the apoptotic pathways in colorectal cancer cells [2, 5].

The extrinsic pathway of apoptosis is induced by the interaction between the ‘death ligands’ and ‘death receptors’, subsequently resulting in the activation of caspase 8. When death ligands, including TRAIL and FAS, bind to the death receptors, such as the TRAIL receptor and CD95, the *d* *eath-* *i* *nducing* *s* *ignalling* *c* *omplex* (DISC)-protein complex is formed. The formation of DISC is followed by the activation of caspase 8, which sequentially activates caspase 3 to initiate apoptosis [3]. Previous studies have shown that the expression of FasL is upregulated in colon cancer [34]. Since the novel nucleoside analogues induced caspase 8 activity in both the HT-29 and Caco-2 cells, it may be reasonable to deduce that the novel nucleosides induced apoptosis via the extrinsic pathway.

Mitochondria play a central role in intrinsic apoptotic signalling [35]. Disruption of the mitochondrial membrane potential (Δψm) and the translocation of cytochrome c from the mitochondria are early markers of apoptosis [36, 37]. Compared to camptothecin, the nucleoside analogues caused a small decrease in the Δψm of both cell lines. Therefore, the major changes observed in the cellular distribution of cytochrome c were unexpected. In the Caco-2 cells, the three test nucleosides 1, 2 and 5 respectively, caused an 8, 8.8 and a 9-fold increase of cytoplasmic cytochrome c levels, respectively, compared to the untreated control. Camptothecin caused the greatest increase in cytosolic cytochrome c, with an 11.2 fold increase above the untreated control. A similar pattern was observed for HT-29 cells; test nucleoside 1, 2 and 5 respectively, caused a 7.4, 8.1 and a 7.7 fold increase in cytosolic cytochrome c compared to the untreated control, but not as high as camptothecin (9.2 fold). Thus, the release of cytochrome c from mitochondria in the HT-29 and Caco-2 cells after treatment with the nucleoside analogues, appears to occur by a mechanism that does not include mitochondrial depolarization. A decrease in mitochondrial membrane potential is thought to be a prerequisite for the release of cytochrome c [38]. Therefore, the results from this study indicate that the two events were not associated. In support of this, there are several reports in the literature that confirm that the release of cytochrome c is not always linked to a change in the mitochondrial membrane potential [37, 39].

In order to better understand the unusual mitochondrial effects, we investigated the expression of Bcl-2 and Bax in both cell lines using immunofluorescence microscopyand, evaluated the expression levels of each of the two proteins, following nucleoside treatment. Bax (Bcl-2-associated X protein) and Bak (Bcl-2-antagonist killer) are two important pro-apoptotic Bcl-2 proteins [40]. Bax is normally present in the cytosol of living cells and translocates to the mitochondria during apoptosis due to the up-regulation of the tumour suppressor protein, p53 [41, 42]. However, in Caco-2 cells, p53 is not expressed [43], whereas HT-29 cells contain a mutated form of p53 (point mutation R273H), which may influence its functionality and its effects on Bax and Bcl-2 [43, 44]. It is therefore possible that the increase in cytosolic cytochrome c is not associated with Bax/Bak mitochondrial pore formation and occurs independently of p53.

Our results indicate that in both cell lines Bcl-2 is strongly expressed and is associated with the nucleus, while Bax showed lower expression levels and is located primarily in the perinuclear space which is consistent with a mitochondrial association. In both cell lines, exposure to the nucleosides did not reveal any marked changes in subcellular location or expression levels of either Bcl-2 or Bax (S5–S8 Figs).

Despite the fact that Bcl-2 is generally believed to be an anti-apoptotic protein, some studies have shown that Bcl-2 can also function in a pro-apoptotic manner [45]. Portier and Taglialatela [46] proposed that the ability of Bcl-2 to function as either pro-or anti-apoptotic is dependent on its subcellular location. Bcl-2 associated with the mitochondrial membrane binds to Bax and prevents the formation of homo-dimers responsible for mitochondrial pore formation [47]. The mechanism and proteins involved in nuclear Bcl-2 mediated apoptosis remains unexplored. This study provides indirect support for the idea that nuclear associated Bcl-2 may be pro-apoptotic. The strong expression of nuclear Bcl-2 combined with the lower expression of Bax offers an explanation for the induction of apoptosis in the absence of mitochondrial membrane depolarisation.

Caspase 8 has been shown to have an important role in the feedback loop of effector caspase activation and cytochrome c release. Bid, a pro-apoptotic Bcl-2 protein, is a substrate of caspase 8. When caspase 8 cleaves Bid, the truncated Bid (tBid) is able to activate Bax to form Bax multimers in the mitochondria, thereby leading to the release of cytochrome c independently, with a resultant change in the mitochondrial membrane potential [48,49,50]. This process, nevertheless still requires a loss of mitochondrial membrane potential and the formation of Bax multimers. The lack of a mitochondrial membrane potential loss and the low Bax expression levels does not support the notion that caspase 8 is responsible for cytochrome c translocation and the activation of the intrinsic apoptotic pathway.

The release of cytochrome c from the mitochondria triggers the activation of caspase 9 in a complex with dATP and Apaf-1 to form the apoptosome. Considering this, we evaluated the ability of the nucleosides and camptothecin to induce caspase 9 activity. Campothecin was able to induce caspase activity, whereas the three test nucleosides were not. The lack of caspase 9 activation by the test nucleosides in both cell lines indicate that the classical intrinsic pathway is not induced by the test nucleosides.

The microscopic evaluation of the changes in cell morphology may however provide a clue to the increased cytoplasmic cytochrome c levels. All three nucleosides were able to induce extensive cytoplasmic vacuole formation, although a slower process for nucleoside 5. Cytoplasmic vacuolisation is not usually associated with apoptosis, but rather with non-apoptotic cell death and/or autophagy [51]. Initial morphological studies indicated the absence of orange/red staining of the vacuoles with acridine orange. Furthermore, their size and perinuclear localisation indicate that they are not likely to be autophagic in nature. In addition, the vacuoles appeared to be fluid filled lacking either cytoplasm or cytoplasmic organelles, as is expected in autophagy.

In order to analyse the nature of the vacuoles, cells were incubated with MDC, a marker for autophagic vacuoles. Chloroquine, a known inducer of autophagic vacuole formation in different cell types, was used as a positive control [17, 18]. MDC accumulates in autophagic vacuoles once the autophagosomes have fused with lysosomes [19, 20, 21]. It also labels acidified endosomes and lysosomes [19]. Nucleoside 2 and chloroquine caused the formation of distinct, highly fluorescent granules. The punctate smaller granules observed with controls, nucleoside 1 and camptothecin are assumed here to be lysosomal in nature. Importantly, the larger highly fluorescent granules did not co-localize exclusively with the vacuoles observed under phase contrast, but rather with the dark granules (Fig 10). This indicates that the vacuolisation induced by these compounds are neither acidic nor autophagic in nature and supports the observations made using acridine orange (Fig 8). In addition the results obtained provide preliminary evidence for the induction of autophagy by nucleoside 2 and possibly nucleoside 5, in both cell lines. Though an investigation of autophagic flux is beyond the scope of the present study it will provide valuable information regarding the type of cell death and the implications of autophagic vacuole formation induced by these compounds. It is of particular interest that despite their similarity of chemical structure, only nucleoside 2 and 5 caused the distinct formation of potentially autophagic vacuoles.

Autophagy, can however explain the increase in cytoplasmic cytochrome c, since mitochondria are degraded in autophagosomes resulting in an increase in cytoplasmic cytochrome c. This would not affect the membrane potential of remaining intact mitochondria and provides an explanation for the change in cytochrome c distribution in the absence of a loss of mitochondrial membrane potential. The ability of camptothecin to induce caspase 9 activity combined with a change in mitochondrial membrane potential and a lack of large granule formation with MDC confirms previous reports that camptothecin is able to cause cell death via the intrinsic apoptotic pathway.

Autophagy, a pro-survival mechanism is not normally associated with apoptotic features like nuclear fragmentation, caspase activation and annexin V binding to phosphatidyl-serine as seen in this study. However, autophagic cell death is well described in the literature. Cytoplasmic vacuolisation is a prominent feature of paraptosis, which includes cytoplasmic vacuolisation, mitochondrial swelling and endoplasmic reticulum stress and annexin V binding to membranes. However, paraptosis is not associated with caspase activation, nuclear fragmentation and the presence of apoptotic bodies, thereby excluding the possibility of the nucleosides causing cell death by discreet paraptosis [52, 53].

Nucleoside 5 effects on actin aggregation is of particular interest and require further investigation. The formation of actin aggregates has implications for cell survival and completion of mitosis [54]. This occurred selectively in HT-29 cells but not in the Caco-2 cells. This is an early event and was typically seen within hours of addition of nucleoside 5 to the cells. It may be of great importance that this occurs in conjunction with apoptosis and potential autophagic cell death. The formation of actin aggregates, combined with its low IC_50_ values and ability to induce apoptosis, identifies nucleoside 5 as a promising lead compound.

This study has shown that the nucleosides induced apoptosis in combination with extensive cytoplasmic vacuole formation. This association, while not a common occurrence, has been described previously [54, 55]. Preliminary investigations indicate that two of the nucleosides are able to cause the formation of autophagosomes. A study on the effects of nucleoside 2 and 5 on autophagic flux will increase the understanding of the interplay between the two major types of cell death, apoptosis and autophagy. Compounds that are able to cause cytoplasmic vacuolisation in addition to inducing apoptosis and autophagic cell death may prove to have a therapeutic advantage, especially since the apoptotic response is compromised in many types of cancer and is associated with resistance as well as therapeutic failure.

## Conclusions

The nucleoside analogue class of cancer drugs are important in the management of cancer and viral infections. The current study showed a time and dose-dependent induction of apoptosis with a series of novel, structurally related nucleoside analogues in both HT-29 and Caco-2 colon cancer cells. Three nucleosides were identified to be significantly cytotoxic (*p* < 0.05) to the HT-29 and Caco-2 cell lines, and were able to induce apoptosis in the cells at micromolar concentrations. Importantly, the nucleosides showed a degree of selective cytotoxicity towards the cancer cell lines, with minimal cytotoxicity against normal unstimulated leukocytes. The proteolytic phase of apoptosis was initiated after a two hour exposure period to these nucleosides. The novel nucleoside analogues were also associated with an increase in cytoplasmic cytochrome c levels, in the absence of a loss of mitochondrial membrane potential. Although possibly mediated by autophagy, this phenomenon, the role of autophagy and autophagic flux in cell death caused by these nucleosides do require further investigation. Additionally, all three nucleosides induced the rapid formation of non-autophagic cytoplasmic vacuoles. Nucleoside 5 treatment resulted in changes in the cytoskeleton of HT-29 cells, causing the aggregation of actin, which may indicate its potential to disrupt the mitosis. The induction of apoptosis is considered to be the main mechanism underlying the therapeutic efficacy of some anticancer drugs. In view of the described cytoplasmic vacuolization, together with apoptosis and potentially autophagy, the nucleoside analogues described here certainly warrant further investigation.

## Supporting Information

S1 Fig Representative dose response curves of nucleoside 1, 2, 5 and camptothecin on HT-29 and Caco-2 cells. Cells were exposed to a concentration range (0.005–120 μM) of nucleosides and camptothecin for 48 hrs and cell viability was determined by the MTT assay.
(TIF)

S2 Fig Representative scattergraphs showing the changes in the mitochondrial membrane potential of HT-29 and Caco-2 cells. Cells were exposed to 100 μM of the nucleosides for 24 hours and stained with JC-1. Untreated HT-29 cells (A), HT-29 treated with camptothecin (B), HT-29 cells treated with nucleoside 5 (C), Caco-2 cells treated with camptothecin (D) and nucleoside 2 (E).
(TIF)

S3 Fig Representative scattergraphs showing typical apoptotic and necrotic HT-29 and Caco-2 cell populations as detected by annexin V-FITC and propidium iodide staining. Cells were exposed to 100 μM of the nucleosides for 24 hours and stained with annexin V and propidium iodide. (A) Untreated HT-29 cells, (B) Untreated Caco-2 cells (C) Caco-2 cells and camptothecin, (D) Caco-2 cells and nucleoside 5, (E) HT-29 cells and nucleoside 1, (F) HT-29 cells and nucleoside 5.
(TIF)

S4 Fig Camptothecin induce caspase 9 activity in contrast with nucleoside 1, 2 and 5. Cells were exposed to 50 μM of test nucleosides and 20 μM of campthecin for 24 hours.
(TIF)

S5 Fig Expression of Bcl-2 in HT-29 cells exposed to the test nucleosides. Cells were exposed to 50 μM test nucleosides for 8 hours.Scalebar: 20 μm.
(TIF)

S6 Fig Expression of Bcl-2 in Caco-2 cells exposed to the test nucleosides. Cells were exposed to 50 μM test nucleosides for 8 hours. Scalebar: 20 μm.
(TIF)

S7 Fig Expression of Bax in HT-29 cells exposed to the test nucleosides. Cells were exposed to 50 μM test nucleosides for 8 hours. Scalebar: 20 μm.
(TIF)

S8 Fig Expression of Bax in Caco-2 cells exposed to the test nucleosides. Cells were exposed to 50 μM test nucleosides for 8 hours. Scalebar: 20 μm.
(TIF)
