# Supplementary figures and images for: Aberrant Apoptotic Response of Colorectal Cancer Cells to Novel Nucleoside Analogues
Source: PLoS One. 2015 Sep 21;10(9):e0138607. doi: 10.1371/journal.pone.0138607 (PMC4577089; doi:10.1371/journal.pone.0138607)

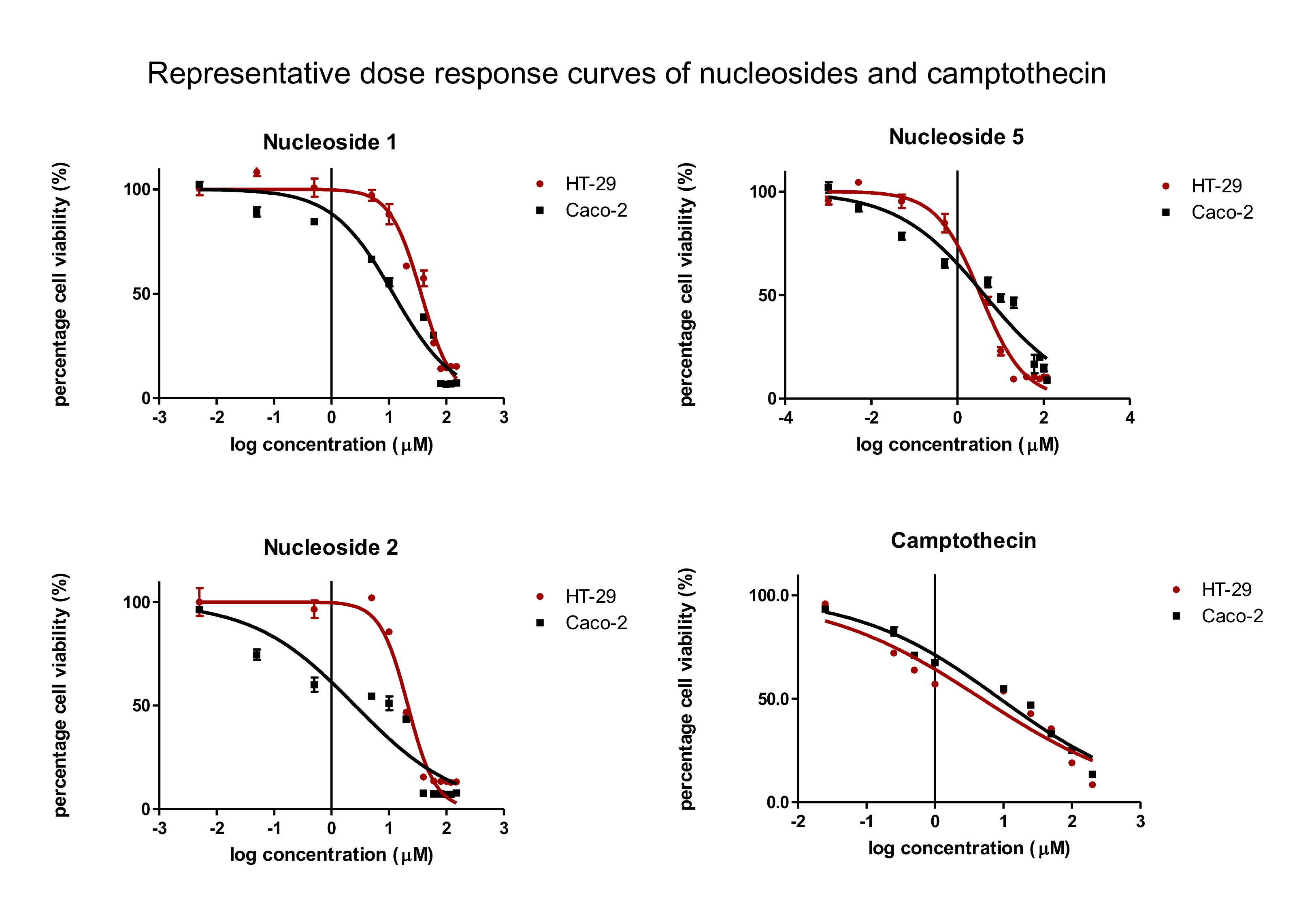

Supplement: S1 Fig — Cells were exposed to a concentration range (0.005–120 μM) of nucleosides and camptothecin for 48 hrs and cell viability was determined by the MTT assay. (TIF) [file pone.0138607.s001.tif]

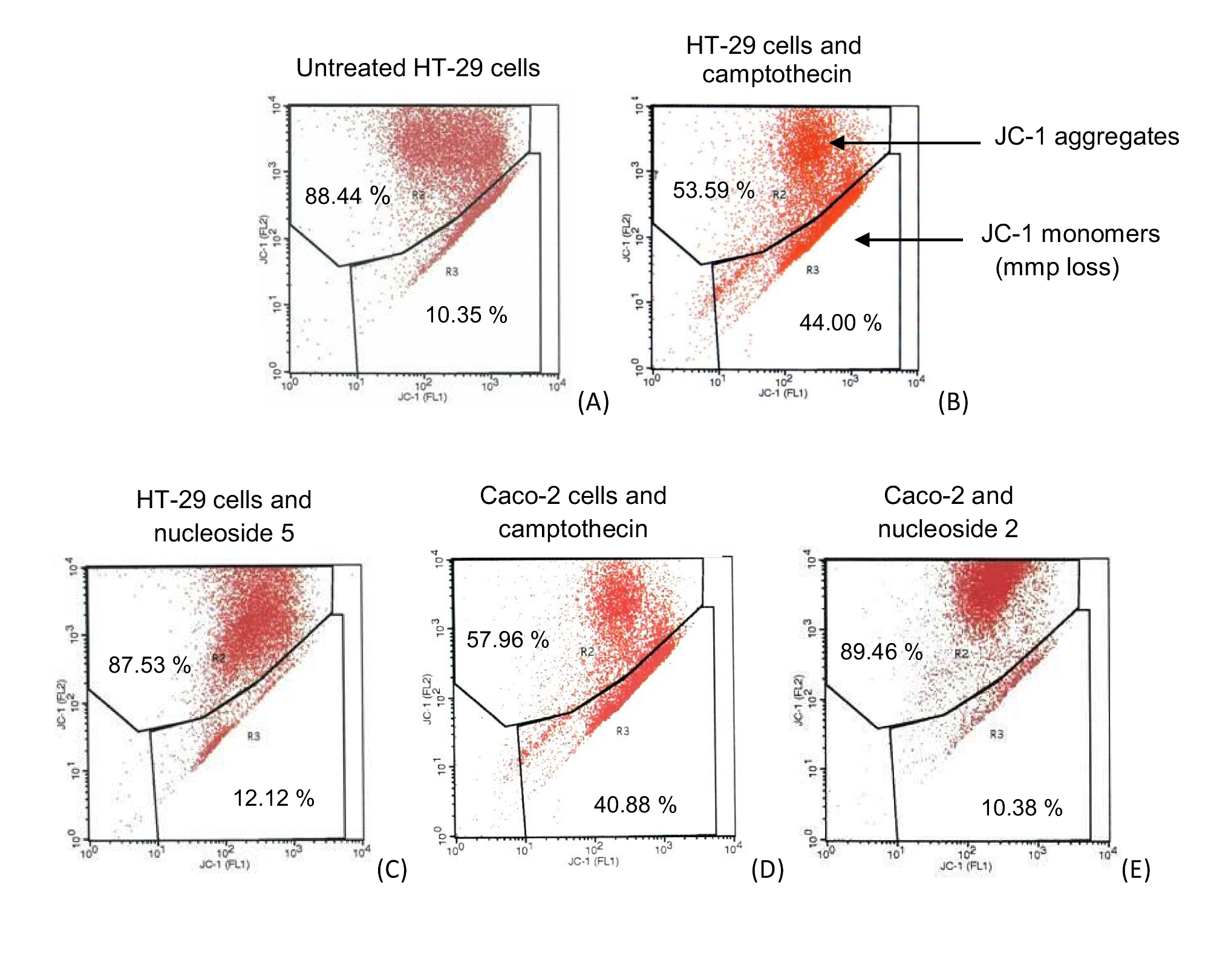

Supplement: S2 Fig — Cells were exposed to 100 μM of the nucleosides for 24 hours and stained with JC-1. Untreated HT-29 cells (A), HT-29 treated with camptothecin (B), HT-29 cells treated with nucleoside 5 (C), Caco-2 cells treated with camptothecin (D) and nucleoside 2 (E). (TIF) [file pone.0138607.s002.tif]

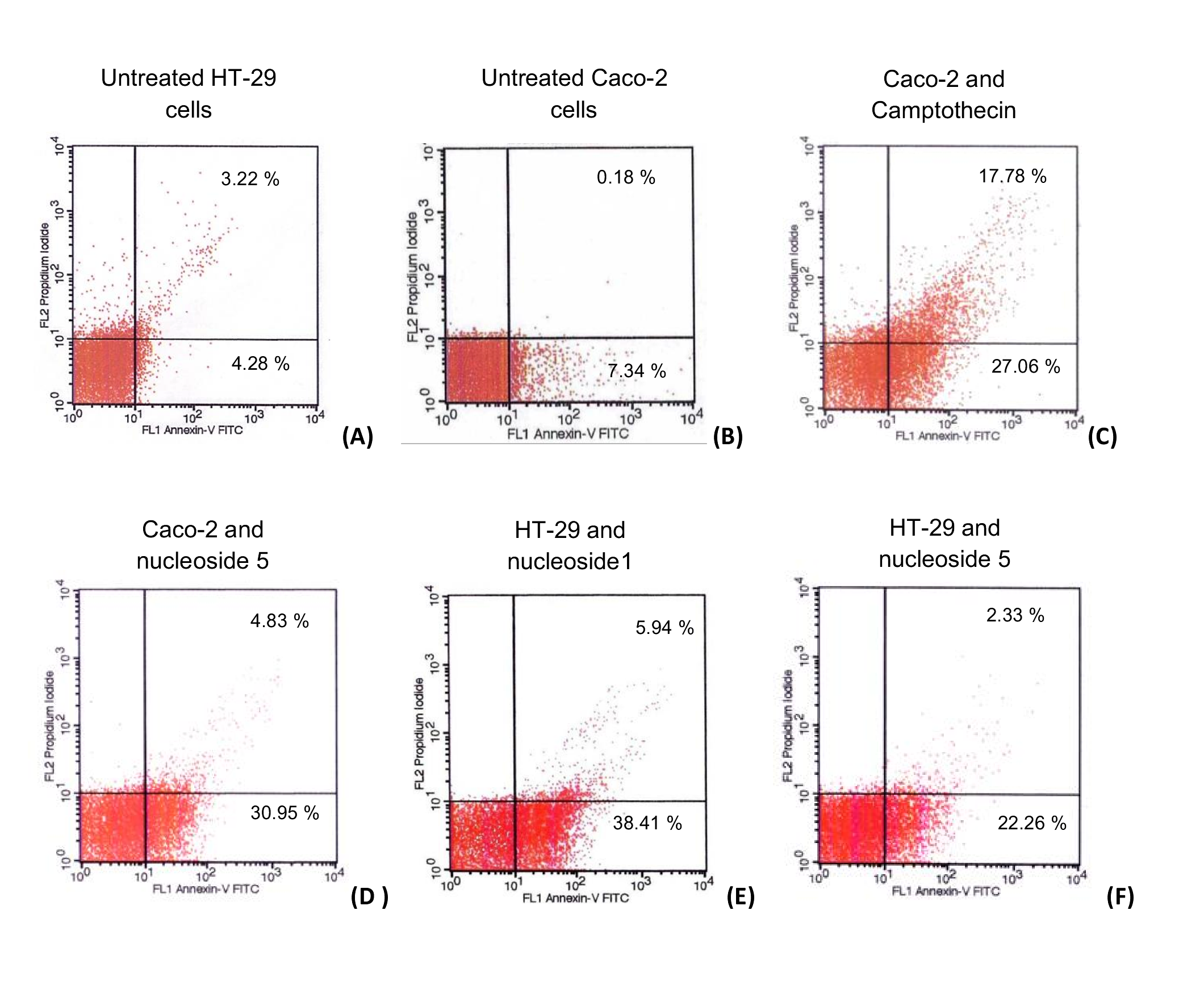

Supplement: S3 Fig — Cells were exposed to 100 μM of the nucleosides for 24 hours and stained with annexin V and propidium iodide. (A) Untreated HT-29 cells, (B) Untreated Caco-2 cells (C) Caco-2 cells and camptothecin, (D) Caco-2 cells and nucleoside 5, (E) HT-29 cells and nucleoside 1, (F) HT-29 cells and nucleoside 5. (TIF) [file pone.0138607.s003.tif]

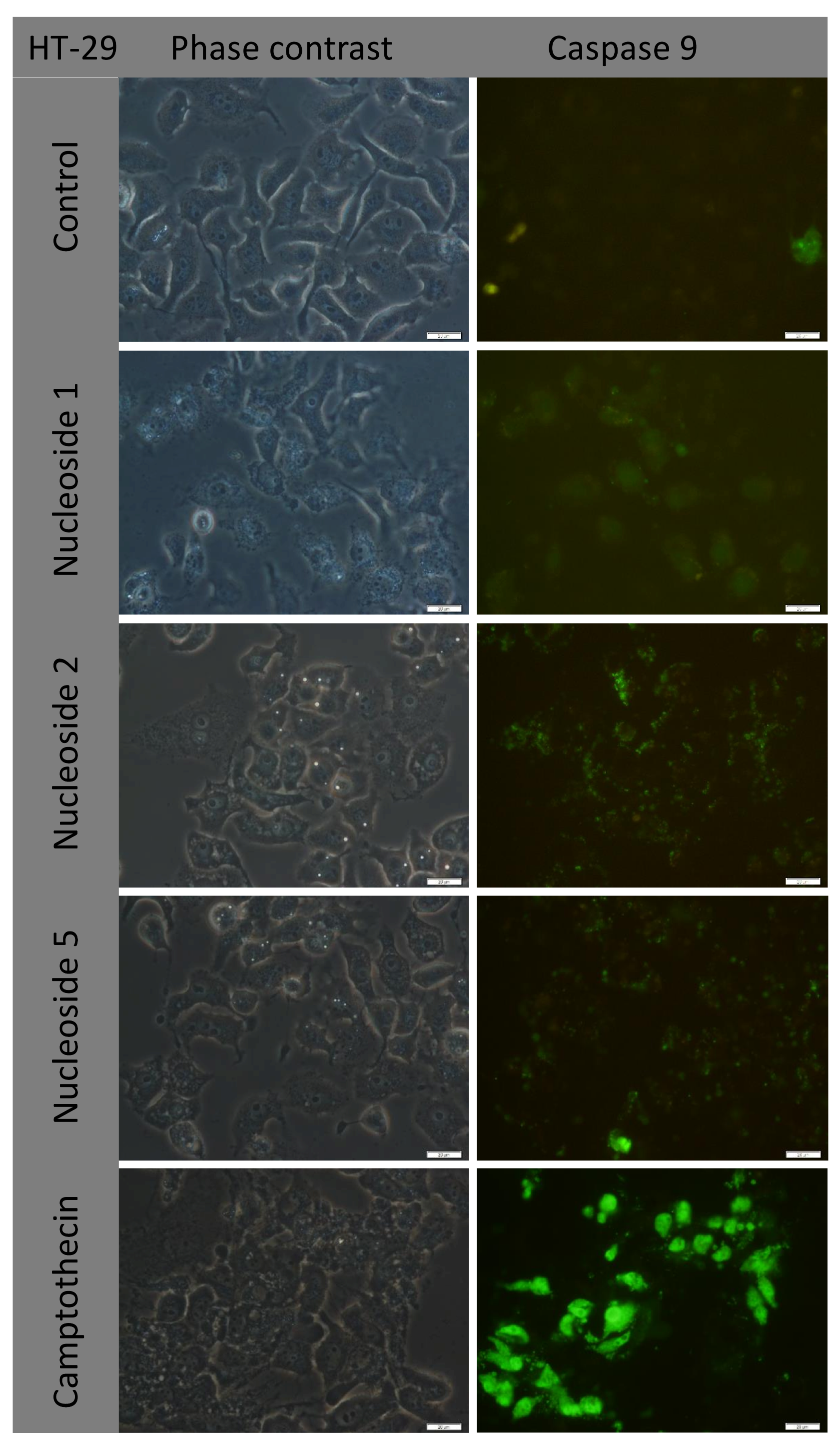

Supplement: S4 Fig — Cells were exposed to 50 μM of test nucleosides and 20 μM of campthecin for 24 hours. (TIF) [file pone.0138607.s004.tif]

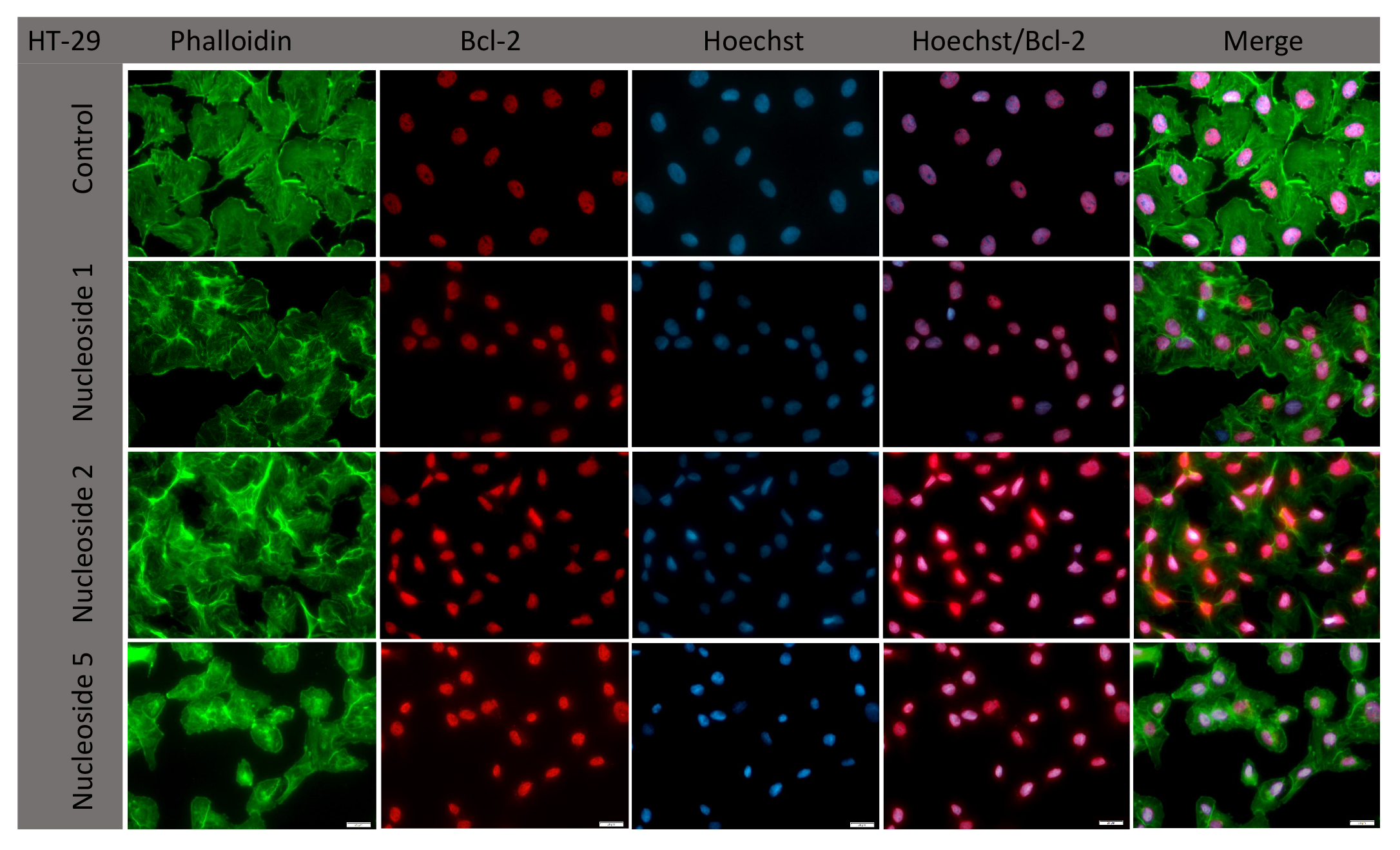

Supplement: S5 Fig — Cells were exposed to 50 μM test nucleosides for 8 hours.Scalebar: 20 μm. (TIF) [file pone.0138607.s005.tif]

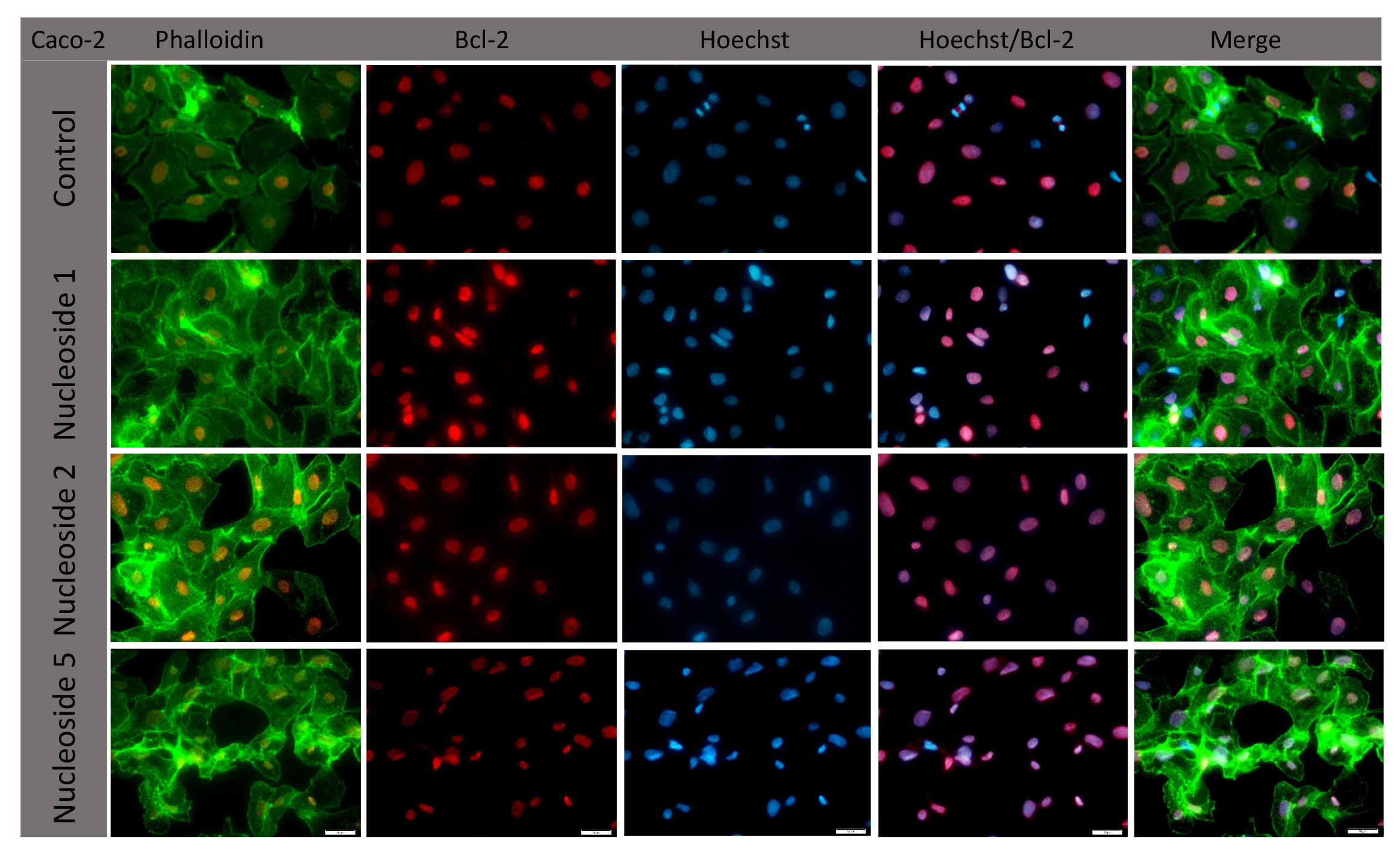

Supplement: S6 Fig — Cells were exposed to 50 μM test nucleosides for 8 hours. Scalebar: 20 μm. (TIF) [file pone.0138607.s006.tif]

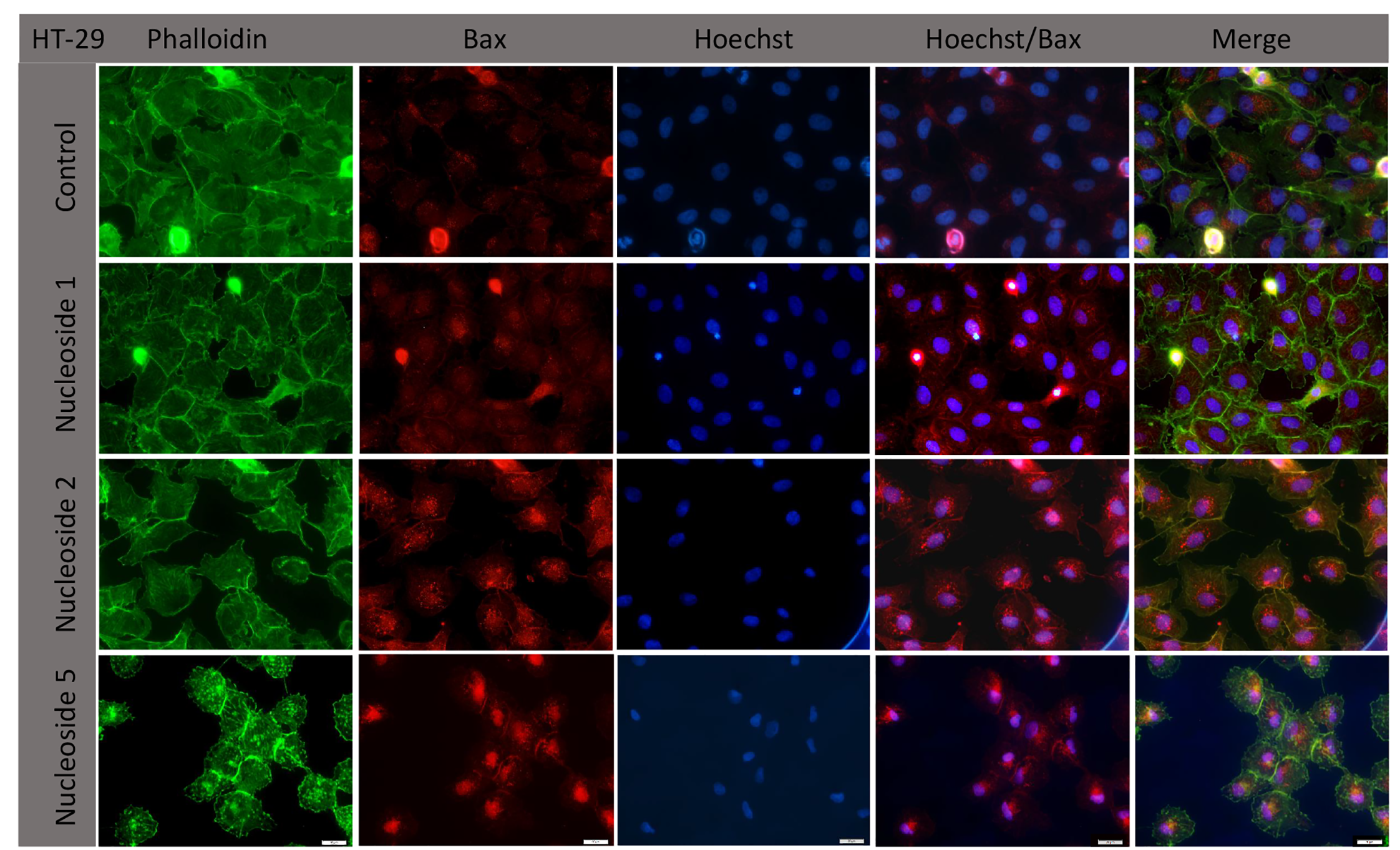

Supplement: S7 Fig — Cells were exposed to 50 μM test nucleosides for 8 hours. Scalebar: 20 μm. (TIF) [file pone.0138607.s007.tif]

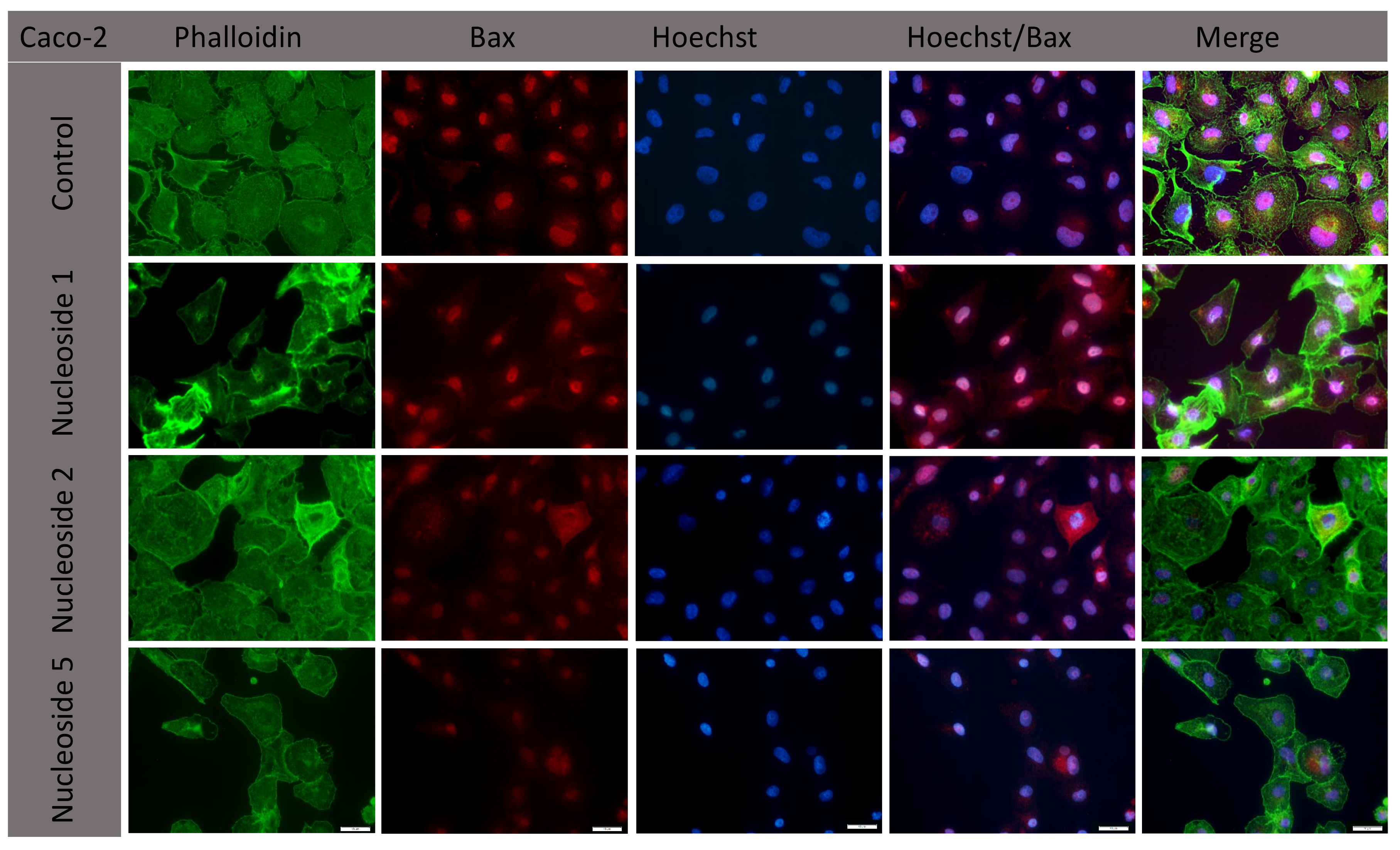

Supplement: S8 Fig — Cells were exposed to 50 μM test nucleosides for 8 hours. Scalebar: 20 μm. (TIF) [file pone.0138607.s008.tif]
